# A Theory- and Evidence-Based Digital Intervention Tool for Weight Loss Maintenance (NoHoW Toolkit): Systematic Development and Refinement Study

**DOI:** 10.2196/25305

**Published:** 2021-12-03

**Authors:** Marta M Marques, Marcela Matos, Elina Mattila, Jorge Encantado, Cristiana Duarte, Pedro J Teixeira, R James Stubbs, Falko F Sniehotta, Miikka Ermes, Marja Harjumaa, Juha Leppänen, Pasi Välkkynen, Marlene N Silva, Cláudia Ferreira, Sérgio Carvalho, Lara Palmeira, Graham Horgan, Berit Lilienthal Heitmann, Elizabeth H Evans, António L Palmeira

**Affiliations:** 1 CIPER -Interdisciplinary Centre for the Study of Human Performance Faculty of Human Kinetics University of Lisbon Lisbon Portugal; 2 Comprehensive Health Research Centre NOVA Medical School Universidade Nova de Lisboa Lisbon Portugal; 3 Center for Research in Neuropsychology and Cognitive and Behavioral Intervention (CINEICC) Faculty of Psychology and Educational Sciences University of Coimbra Coimbra Portugal; 4 VTT Technical Research Centre of Finland Ltd Espoo Finland; 5 School of Education, Language and Psychology York St John University York United Kingdom; 6 Population Health Sciences Institute Faculty of Medical Sciences Newcastle University Newcastle Upon Tyne United Kingdom; 7 NIHR Policy Research Unit Behavioural Science Newcastle University Newcastle Upon Tyne United Kingdom; 8 Faculty of Behavioural, Management and Social Sciences University of Twente Twente Netherlands; 9 Centro de Investigação em Desporto, Educação Física e Saúde Universidade Lusófona de Humanidades e Tecnologias Lisbon Portugal; 10 HEI-Lab: Digital Human-Environment Interaction Lab School of Psychology and Life Sciences Lusófona University Lisbon Portugal; 11 Biomathematics and Statistics Scotland Aberdeen United Kingdom; 12 Research Unit for Dietary Studies, The Parker Institute Bispebjerg and Frederiksberg Hospital The Capital Region Denmark; 13 Department of Public Health Section for General Practice University of Copenhagen Copenhagen Denmark; 14 Department of Psychology Durham University Durham United Kingdom

**Keywords:** mHealth, behavior change techniques, weight management, motivation, self-regulation, emotion regulation, self-monitoring, user testing, logic models

## Abstract

**Background:**

Many weight loss programs show short-term effectiveness, but subsequent weight loss maintenance is difficult to achieve. Digital technologies offer a promising means of delivering behavior change approaches at low costs and on a wide scale. The Navigating to a Healthy Weight (NoHoW) project, which was funded by the European Union’s Horizon 2020 research and innovation program, aimed to develop, test, and evaluate a digital toolkit designed to promote successful long-term weight management. The toolkit was tested in an 18-month, large-scale, international, 2×2 factorial (motivation and self-regulation vs emotion regulation) randomized controlled trial that was conducted on adults with overweight or obesity who lost ≥5% of their body weight in the preceding 12 months before enrollment into the intervention.

**Objective:**

This paper aims to describe the development of the NoHoW Toolkit, focusing on the logic models, content, and specifications, as well as the results from user testing.

**Methods:**

The toolkit was developed by using a systematic approach, which included the development of the theory-based logic models, the selection of behavior change techniques, the translation of these techniques into a web-based app (NoHoW Toolkit components), technical development, and the user evaluation and refinement of the toolkit.

**Results:**

The toolkit included a set of web-based tools and inputs from digital tracking devices (smart scales and activity trackers) and modules that targeted weight, physical activity, and dietary behaviors. The final toolkit comprised 34 sessions that were distributed through 15 modules and provided active content over a 4-month period. The motivation and self-regulation arm consisted of 8 modules (17 sessions), the emotion regulation arm was presented with 7 modules (17 sessions), and the combined arm received the full toolkit (15 modules; 34 sessions). The sessions included a range of implementations, such as videos, testimonies, and questionnaires. Furthermore, the toolkit contained 5 specific data tiles for monitoring weight, steps, healthy eating, mood, and sleep.

**Conclusions:**

A systematic approach to the development of digital solutions based on theory, evidence, and user testing may significantly contribute to the advancement of the science of behavior change and improve current solutions for sustained weight management. Testing the toolkit by using a 2×2 design provided a unique opportunity to examine the effect of motivation and self-regulation and emotion regulation separately, as well as the effect of their interaction in weight loss maintenance.

## Introduction

### Background

Behavior change interventions for overweight and obesity that target dietary behaviors, physical activity, and other weight management components show beneficial effects in reducing weight and improving health, at least in the short term [1,2], but most individuals experience weight regain over subsequent months and years [3,4]. Thus, a key challenge for such interventions is to find sustainable and scalable methods for long-term weight loss maintenance (WLM) [5,6]. The emergence and rapid growth of digital behavior change interventions (ie, sets of activities or products designed to change specified behavior patterns through digital technology) are a response to the urgent need for scalability and sustainability and can help to better understand the complexity behind individuals’ decisions and engagement in behaviors that affect their health and well-being, including sustained weight management [1,7].

Although research on the effectiveness of digital behavior change interventions is at an early stage, existing reviews have found small effects and between-study variability on outcomes and behaviors [8,9]. These interventions can be optimized by identifying the features that contributed to their effectiveness through systematic theory- and evidence-based processes of intervention development, implementation, testing, and reporting (eg, Medical Research Council [10] and Template for Intervention Description and Replication guidance [11]).

In the context of weight management, the core features of effective interventions include techniques in line with self-regulation theories, such as goal-setting, self-monitoring of weight and behavior, feedback on behavior and weight, and plans to cope with risk factors for weight regain and relapse prevention (eg, problem-solving) [12-15]. Autonomous motivation has also been associated with change in energy balance behaviors for obesity management [16]. As the number of digital interventions based on motivation and self-regulation approaches to sustained weight management is limited [17], it is important to test if these theoretical approaches produce sustained behavior changes in the context of WLM. In addition, contextual-based emotion regulation strategies that promote compassion, acceptance, and mindfulness [18-20] may also have an impact on behavior changes that promote WLM [21]. With these considerations in mind, we developed a theory-based toolkit, delivered as a portfolio of embedded digital tracking technologies (smart scale and activity trackers), mini apps, and web resources accessible through a computer, tablet, and mobile phone, as part of the European Union’s Horizon 2020 project *NoHoW: Evidence-Based Tools for Weight Loss Maintenance*.

**Figure 1:**
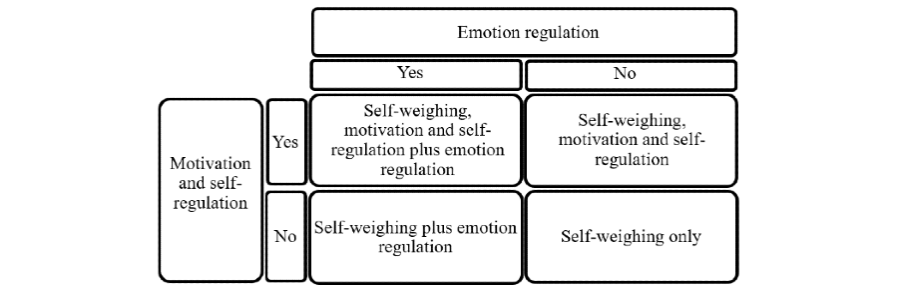
The Navigating to a Healthy Weight trial design.

The toolkit was used in the international Navigating to a Healthy Weight (NoHoW) innovative, 2×2 factorial, single-blind randomized controlled trial (RCT) for WLM. All 4 arms of the intervention included self-weighing and self-monitoring of activity using commercial Wi-Fi scales and activity trackers. The active control arm included access to generic weight management content, and the 3 intervention arms consisted of *motivation and self-regulation* content, *emotion regulation* content, and *combined motivation and self-regulation*+*emotion regulation* content (Figure 1; detailed information about the NoHoW project and trial can be found elsewhere [22]).

### Aim

This paper aims to describe the processes involved in the development of the NoHoW Toolkit to a stage appropriate for an RCT evaluation (trial registration number: ISRCTN88405328) and provide a detailed overview of the NoHoW Toolkit.

## Methods

### Systematic and Iterative Process

The development of the NoHoW Toolkit involved a multidisciplinary team of researchers in behavioral science, obesity and weight management, exercise and nutrition, software development, data analytics, biomathematics, and user experience (UX). It followed a systematic and iterative process incorporating both theory- and evidence-based approaches, as well as user testing to refine usability, and it also followed best practices by conducting the steps that are commonly recommended by the Behaviour Change Wheel [23], the intervention mapping approach [24], and the Medical Research Council [25] guidance for intervention development. It consisted of the following five steps: (1) development of theory-based logic models; (2) selection of the content, that is, behavior change techniques; (3) translation of these techniques into a coherent web-based app (NoHoW Toolkit components); (4) technical development; and (5) user evaluation and refinement of the toolkit for subsequent evaluation in the context of an RCT.

### Step 1: Development of Theory-Based Logic Models

The selection of the psychological and behavioral factors to be targeted by the toolkit was based on existing studies on the most relevant theoretical frameworks in the context of physical activity, healthy eating, and weight management [2]. These studies were reviewed by the core research team and informed the NoHoW theoretical logic models that were developed. These logic models schematically represent the relationships among (1) the primary and secondary outcomes, including behavioral outcomes; (2) the theoretical mediators that were hypothesized to explain the effect of the toolkit content on the primary and secondary outcomes; (3) the content of the toolkit; and (4) the hypothesized moderators of the intervention effects. This task was executed by the behavioral science team with feedback from the members of the larger project consortium and the external advisory board.

### Step 2: Selection of Behavior Change Techniques

The next developmental task was the identification and selection of the specific techniques that would form the content of the NoHoW Toolkit. This selection was derived from the studies reviewed (step 1) describing the behavior, motivation, and emotion regulation techniques hypothesized to have an impact on the theory-based mediators of WLM as represented in the logic models. For example, providing choice is a core technique to foster autonomy. We conducted an additional scoping review to identify the frequently used and effective intervention techniques and modes of delivery used in digital behavior change interventions for changing health behaviors in the context of long-term weight management (Multimedia Appendix 1). The selection of the final set of techniques was carried out through discussions among the team members. To ensure standardization in the description of the NoHoW Toolkit, the selected techniques were reported using the identifiers and labels from reliable taxonomies (when available) such as the Behavior Change Techniques Taxonomy (BCTT) version 1 (eg, BCTT 1.2 Problem Solving) [26] or the classification of Motivation and Behavior Change Techniques (MBCTs; eg, MBCT 6: Providing Choice) [27]. No taxonomy is yet available for the emotion regulation techniques.

### Step 3: Translation of the Techniques Into the NoHoW Toolkit Components

Table 1 presents an overview of the development tasks (steps 3 to 5). Initially, the research team created user cases involving personas based on recent research identifying individuals involved in WLM [28] (who varied in age, gender, weight history, digital literacy, and reasons for participating in a WLM intervention) and scenarios (eg, first visit, throughout the intervention, and during a relapse) to describe the target users and their potential experiences when using the toolkit. This exercise provided the starting point for specifying the functional requirements of the toolkit and identifying how the techniques selected in step 3 would be implemented and also facilitated the development of a common language between the content development and technology development partners. A guidance manual was created to aid the teams involved in the development of the modular content of the toolkit. The manual stipulated that the content of each module should identify (1) specific behavior goals; (2) theoretical constructs targeted; (3) techniques targeted; (4) rationale: how the goal, theoretical construct, and techniques are linked; and (5) the mode of delivery of each technique (eg, video or text) described in a way that would inform their implementation by the software developers and designers. Scripts were developed for each implementation.

**Table 1:** NoHoW Toolkit^a^ development process.

Tasks	Team responsible
Task 1: persona and scenario development	Behavior change team and UX^b^ team
Task 2: development of the overall content of the toolkit (eg, sessions) based on the motivation, self-regulation, and emotion regulation theories of behavior change	Behavior change team
Task 3: system architecture design	Software development team
Task 4: full description of each implementation (technique or clusters of techniques) for each content feature (eg, session)	Behavior change team
Task 5: development of a list of possible modalities in which the techniques would be implemented (eg, quiz, animation video, audio, text, and testimonial)	Behavior change team
Task 6: feedback about feasibility of implementation, engagement, and other options and details of the technical implementation	Software development team
Task 7: adjustments in accordance	Behavior change team
Task 8: UI^c^ design	UX team and software development team
Task 9: functional description	Software development team
Task 10: programming	Software development team
Task 11: feasibility study plan	UX team
Task 12: toolkit sessions upload using a content management system	Behavior change team
Task 13: the implementation is user tested	Behavior change team and software development team
Task 14: final adjustments	Behavior change team and software development team

^a^NoHoW: Navigating to a Healthy Weight.

^b^UX: user experience.

^c^UI: user interface.

### Step 4: Toolkit Technical Development

We identified that the 2 most important functionalities of the toolkit were (1) providing the WLM intervention in 3 theoretically informed, evidence-based versions and a control version and (2) supporting the digital tracking of weight, physical activity, and sleep. Given that the toolkit was intended to be used for several months, a positive user interaction and an attractive user interface (UI) were considered necessary. A UX designer was involved in the iterative development of the most crucial UI elements. The design process started with scenario development and identification of the user requirements for the toolkit based on the scenarios, informing the development of the initial UI concepts. The concepts were visualized by the UX designer and tested in small-scale interviews with participants who were similar to the target users. The UI for the intervention sessions was designed through collaboration between the behavior change team and the intervention designers. An admin UI was developed to serve the needs of the intervention designers and trial managers. The intervention designers needed to be able to add, refine, and update content continuously during the toolkit development as well as to define the content, rules, and schedules for different email prompts and reminders to be sent to the toolkit users. The trial managers needed to create new users of the toolkit, assign them to different intervention arms, and manage their status depending on whether they were still involved in the study.

### Step 5: User Evaluation and Toolkit Refinement

We conducted 2 user evaluation studies. The first, which sought to identify key technical and UX-related issues and results from this study, informed the refinements to the NoHoW Toolkit version 1.0. This study was conducted in the United Kingdom with English-speaking adults, recruited primarily by advertisement or invitation from the University of Derby. The inclusion criteria were as follows: aged ≥18 years, able to travel to the University of Derby, able to follow written and verbal information in English, ability to access the internet, and currently overweight or have been overweight in the past, with at least one weight loss attempt. Approval was obtained from the research ethics committee of the University of Derby. A mixed methods approach was used for the analysis of the data through questionnaires, interviews, toolkit use log data, and data from wireless scales and activity trackers (Fitbit [Fitbit LLC]; see Multimedia Appendix 2 for the study description).

After refinement of the toolkit version 1.0 into version 2.0 and translation from English into Portuguese and Danish (languages of the 3 countries where the NoHoW trial was conducted), we conducted a second usability testing study with the Portuguese sample. This was a qualitative study consisting of in-depth interviews, with the aim of understanding users’ motivation to engage with the toolkit and determining which functionalities of the toolkit could promote user engagement (see Multimedia Appendix 3 for the full description). In these interviews, conducted after changes were implemented on top of toolkit version 1.0, the participants were shown a beta version of the toolkit, which included a demonstration of the navigation procedures, and 1 session of the toolkit. The interviews, which lasted approximately 1 hour, were audio-recorded and transcribed verbatim. Thematic analysis was conducted using MAXQDA version 12 (VERBI GmbH). Approval was obtained from the research ethics committee of the University of Lisbon.

## Results

### Step 1: Development of Theory-Based Logic Models

Available evidence from behavioral weight management interventions [17] showed some support for the effectiveness of interventions based on self-regulation theories [13,14,29] and self-determination theory [16]. There is also increasing evidence showing the impact of emotions in weight management [30-34] and indicating that contextual behavioral approaches can contribute to psychological well-being and weight management through the development of skills aimed at reducing the automaticity of maladaptive patterns of behavior (eg, mindfulness, acceptance, and compassion toward internal negative states) [34-38].

The available evidence formed the basis for the NoHoW research hypotheses that WLM could be supported by strategies that promote (1) self-regulation (setting optimal goals and reviewing them, action and coping planning, and action control) and motivation factors (promotion of autonomous motivation vs controlled motivation, intrinsic goals, and flexible regulation to change behaviors and maintain weight loss), (2) emotion regulation factors that may undermine self-management of energy balance–related behaviors (reduce weight-related shame and self-criticism, reduce difficulties in emotion regulation, and increase psychological flexibility, mindfulness, and compassion) and (3) interactions between (1) and (2).

**Figure 2:**
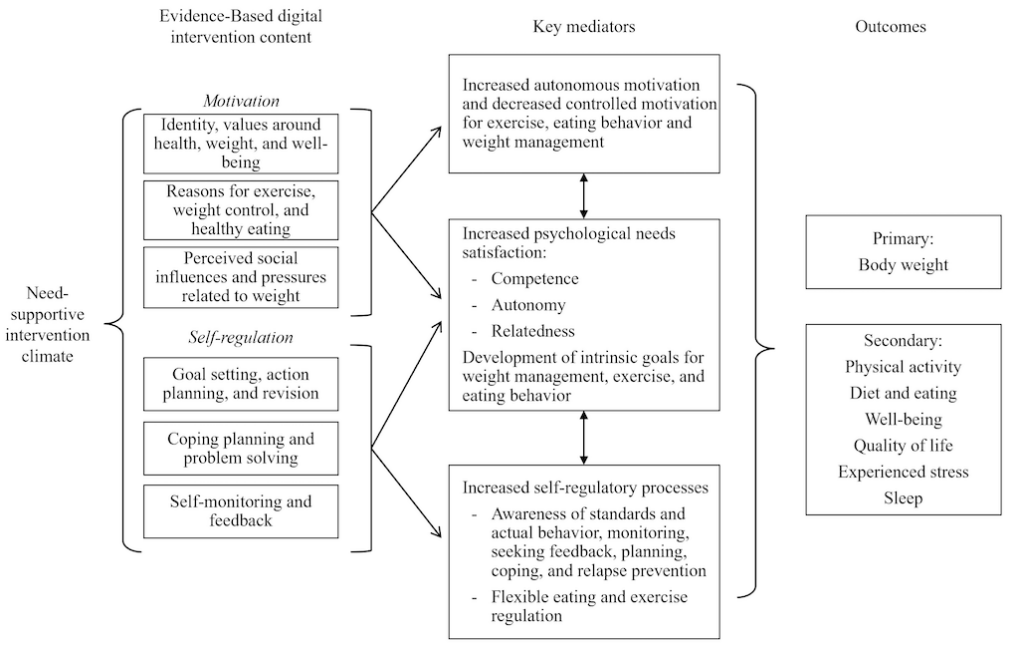
Logic model: motivation and self-regulation arm.

**Figure 3:**
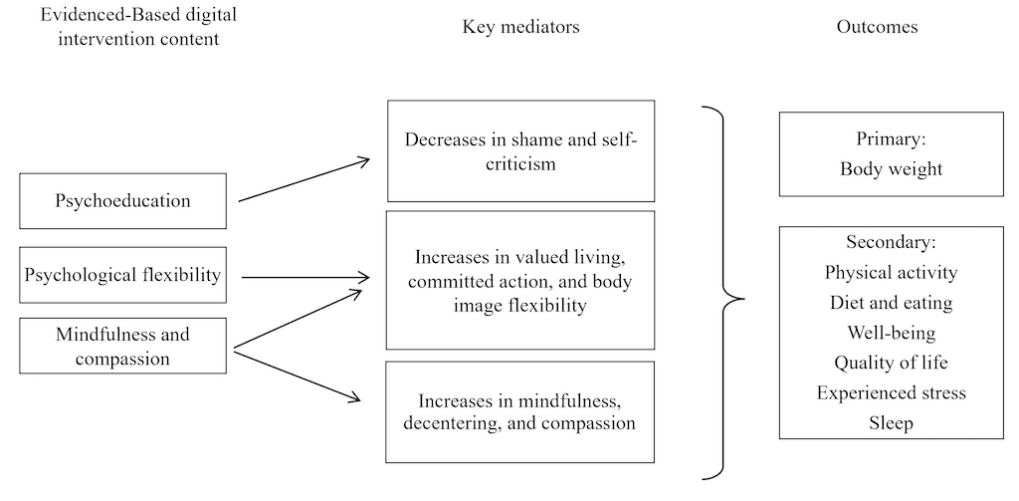
Logic model: emotion regulation arm.

**Figure 4:**
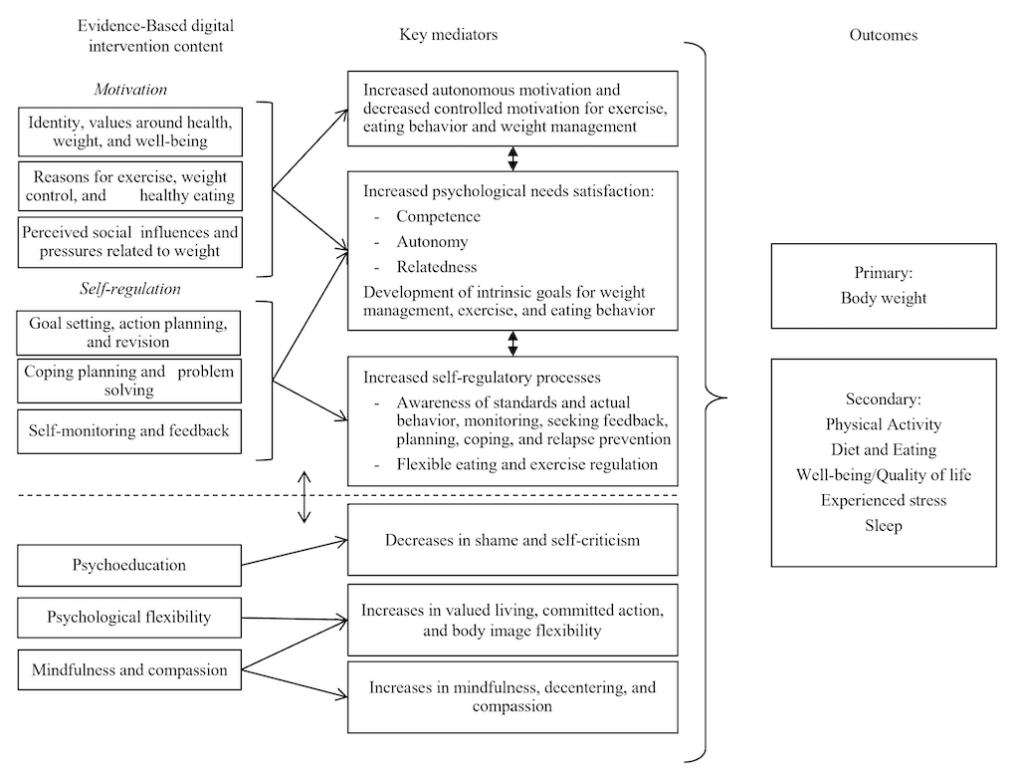
Logic model: combined arm.

The NoHoW logic models, presented in Figures 2-4, schematically represent the relationships derived from these theoretical approaches. We developed 3 logic models, one for each of the intervention arms: *motivation and* *self-regulation* arm, *emotion regulation* arm, and the *combined* arm.

### Step 2: Selection of Behavior Change Techniques

For the *motivation and* *self-regulation* version of the toolkit, 29 intervention techniques were selected: 11 from the BCTT version 1, mainly focusing on goals, planning, and self-monitoring, and 18 from the classification of MBCTs, targeting autonomy, relatedness, and competence (see Multimedia Appendix 4 [26,27] for the full list of techniques). For the *emotion regulation* arm, 25 techniques were defined based on contextual behavioral science approaches, specifically, compassion-focused therapy, mindfulness-based interventions, and acceptance and commitment therapy (see Multimedia Appendix 5 for the full list of techniques).

### Step 3: Translation of the Techniques Into the NoHoW Toolkit Components

#### Overview

**Figure 5:**
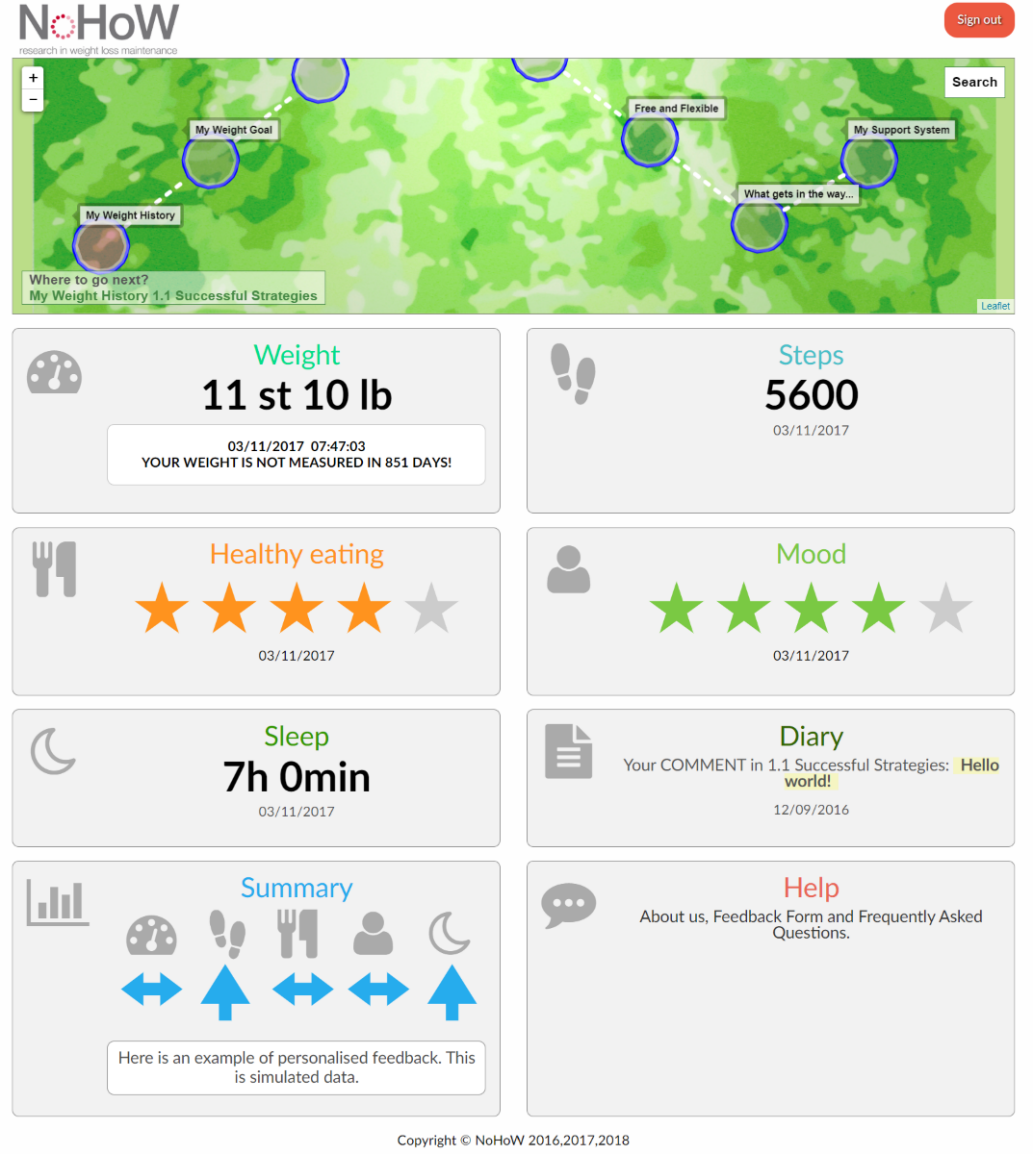
Navigating to a Healthy Weight Toolkit dashboard. st: stone; lb: pounds; h: hours; min: minutes

The implementations of the techniques in the toolkit components are described below. When a user logged in to the toolkit, the main view (Figure 5) consisted of a tile-based dashboard, which was available for all toolkit users; a personal route map, which was only available for intervention users; weekly emails; a diary section (list of comments or reflections that a user could add at the end of each session); and a help section (information about the study, frequently asked questions, and instructions for navigating in the toolkit). The first 3 are described in detail next.

#### Tile-Based Dashboard

The dashboard tiles provided an at-a-glance view of the user’s most recent data. The toolkit presented separate tiles on physical activity, sleep, and weight data retrieved from the Fitbit activity tracker (Charge 2) and the Fitbit smart Wi-Fi scale (Aria). All users could visualize their data for 1 week and for 1 month, 3 months, and 6 months. For the *motivation and* *self-regulation* arm and for the *combined* arm, the tiles also presented the user’s goals and action plans as well as coping plans related to physical activity and diet. These plans could be reviewed at any time in the tiles. In addition, there were 2 tiles for mood and diet, each based, respectively, on the response to a single question, “How do you rate your mood, at the moment?” and “How satisfied are you with your diet, at the moment?” (5-star response scales). The tiles highlighted if no recent data existed, thus prompting continuation of monitoring. By clicking on a tile, the user could view long-term graphical feedback in different time scales and make self-assessments with a star rating feature (Multimedia Appendix 6).

#### Personal Map

The personal map (not available to the control arm) contained the modules of the toolkit, expressed in sessions (Multimedia Appendix 7). The sessions available depended on the arm to which the user was randomized. As the full content for each arm of the toolkit was available from the first day of use, it was considered useful to have a map to guide the user through the optimal theory-informed order of the content. When a user clicked on a module, the sessions of a module were displayed (Multimedia Appendix 7). There were regular sessions (lasting approximately 5 minutes) and short sessions (lasting 1 minute each), and the user was prompted to access a specific activity during the week. The *motivation and self-regulation* arm presented 17 regular sessions, organized in 8 modules. The estimated duration of these sessions ranged from 3 to 19 minutes. There were also 4 short sessions. The *emotion regulation* arm presented 17 regular sessions as well as 2 short ones distributed through 7 modules. The estimated duration ranged from 2 to 27 minutes. The *combined* arm presented all sessions from both arms distributed in 17 weekly modules.

Tables 2 and 3 describe the modules’ sessions for all trial arms. The techniques selected were implemented in the sessions using various types of activities, or implementations, which are described in brief here (Multimedia Appendix 8):

1. Whiteboard animations: 1-minute videos that use animation to provide educational content. The toolkit presented videos about self-monitoring, self-confidence, myths and facts, flexibility and body image, autonomous motivation, evolutionary perspective of eating, conflicting messages, food functions, shame and self-criticism, obstacles to a healthier life, compassion, and mindfulness (Multimedia Appendix 9).
2. Questionnaires: interactive exercises to collect data from the user and provide immediate feedback (eg, a brief questionnaire concerning the reasons for personal ideal weight [motivation and self-regulation arm] or concerning compassionate attitudes [emotion regulation arm]; (Multimedia Appendix 10).
3. Testimonials: short personal stories describing, for example, the difficulties, challenges, and successes in a weight management process (Multimedia Appendix 11). Users could choose between a story of a male or female character.
4. Downloadable audios: audios containing mindfulness exercises, lasting approximately 5 minutes. They were only available in the emotion regulation and combined arms.

**Table 2:** Theme and goals of each module of the motivation and self-regulation arm.

Module (theme)	Core goals	Mechanisms of action
Module 1: My weight history	Review weight change history: weight trajectory and characteristics of previous weight changes (strategies and feelings associated) Identify weight loss strategies or approaches used in the last weight loss attempt and assess their sustainability in time	Self-awareness and competence
Module 2: My weight goal	Learn about self-monitoring and self-referenced feedback and its role in goal-setting and revision, and reflect on individual options and preferences Reflect on ideal and acceptable weights (and where these terms originated and what they mean to the person) Understand the importance of setting self-relevant and optimal goals: set weight-related goal	Self-regulation capacity, competence, autonomy (ownership), and intrinsic (vs extrinsic) goals
Module 3: Myths and facts	Promote factual knowledge about energy balance–related behaviors (exercise and diet) and WLM^a^	Competence and autonomous motivation
Module 4: My healthy goals	Promote awareness of multiple choices around behavior changes and WLM in the long term (there is no “right” way) Find individual interests and seek enjoyment and personal meaning around health behaviors Explore personal resources for engaging in health behaviors (eg, skills)	Autonomy (perceived choice), autonomous motivation, competence, and self-regulation capacity
Module 5: My goals and values	Prompt reflection on personal reasons for WLM (weight goals) and related behaviors by differentiating internal (autonomous) and external (controlled) motives and their relationship to sustained behavior change (sense of ownership) Explore sources of body image or ideal (eg, societal norms, media, and significant others) and its consequences (motivation and well-being)	Intrinsic (vs extrinsic) goals (ie, life aspirations), autonomous versus controlled motivation, autonomy (ownership), and perceived social influences and pressures related to weight
Module 6: Free and flexible	Identify functional and dysfunctional investment in body appearance (by exploring sources of body image or ideal (eg, societal norms, media, and significant others) and its consequences (motivation and well-being) and promote satisfaction with one’s body, at any size Explore links between internal (feels free and choiceful) and external (feels pressured) motives and eating and exercise regulation (eg, rigid vs flexible approach)	Intrinsic (vs extrinsic) goals, autonomous versus controlled motivation, autonomy (ownership), and rigid versus flexible behavior regulation
Module 7: What gets in the way	Identify challenges and barriers to current behavioral patterns and identify resources to increase capability to deal with them Identify strategies to deal with these barriers (coping plans) and to focus attention on how behavior changes serve other important life goals	Competence, autonomous motivation, and self-regulation capacity
Module 8: My support system	Identify sources of social support and reflect on what they mean (eg, pressured and conditional support vs unconditional support) Increase skills in seeking social support and dealing with social and peer pressures (eg, assertiveness) Explore reaching out to others as a role model or source of support and expertise in WLM	Relatedness, autonomous motivation, perceived social influences and pressures related to weight, and self-regulation processes

^a^WLM: weight loss maintenance.

**Table 3:** Theme and goals of each module of the emotion regulation arm.

Module (theme)	Core goals	Mechanisms of action
Module 1: Why do we eat?	Promote the understanding of the difficulties in regulating eating behavior because our systems are not yet evolved to restrict eating behavior (it is not our fault) Promote the knowledge about how our body works to stop people fighting against their bodies and begin working with them instead Promote the understanding that food has multiple functions (it is not our fault) Promote the reflection about the conflicting messages in our modern society about eating and physical activity and using food as a way of comfort and achieving a thin and fit body image Clarify how these conflicting messages create additional stress and how eating may emerge as a way to soothe the self and manage stress	Evolutionary approaches to eating behavior and physical activity, deshaming, and emotion regulation
Module 2: Eating awareness	Unveiling emotional and stress eating Identify the traps in using food to regulate emotions and cope with stress Learn how to eat mindfully Promote awareness of satiety and hunger cues	Emotional eating, emotion regulation, and mindfulness
Module 3: Roadblocks to change	Do we need shame and self-criticism to manage our weight? Understand the evolved functions of shame and self-criticism Clarify the negative effects of self-criticism and shame on weight management, body image, and physical activity Consolidate the inefficacy of shame and self-criticism to cope with stress and maintain changes Promote creative hopelessness	Shame, self-criticism, creative hopelessness, and stress management
Module 4: Living a healthy life	Foster creative hopelessness Promote the clarification of values Identify how life can be so disconnected from values Identify the obstacles to a valued life Discuss the role of avoidance and the control agenda as obstacles to a valued life Create value-related goals and step-by-step actions Encourage committed actions to values in daily life (ie, the importance of healthy eating patterns and physical activity)	Creative hopelessness, values, avoidance, control agenda, and committed action
Module 5: Learning to just be	Introduce and reduce automatic pilot Reflect on the needed shift from the doing mode to the being mode Clarify what mindfulness is and evidence of its benefits Increase awareness and acceptance of the present moment Use the breath as an anchor for the present moment Introduce the 3-minute breathing space as a way of being fully present with a different frame of mind Learn how to use the 3-minute breathing space to deal with difficult emotions, sensations, thoughts, or the stress of daily life Learn how to use the body as an anchor to the present moment experience Increase awareness and acceptance of unwanted internal experiences (emotions and physical sensations) Increase awareness of the body and the body in movement Use mindfulness to take better care of your body Increase awareness and acceptance of thoughts Promote a defused perspective on thoughts: Thoughts are not facts Promote an observer perspective of internal experiences Promote the identification and awareness of stress responses in the body Foster a more adaptive and healthy way to cope with stress and negative emotions	Mindfulness, automatic pilot, being mode, acceptance, awareness, coping, bodily awareness, mindful movement, decentering, and emotion regulation
Module 6: Cultivating compassion	Introduce loving-kindness Understand how the need for compassion emerges from our evolved brain and emotional systems Understand what compassion is Clarify that compassion takes courage Learn the basic skills of cultivating compassion Learn how to prepare the body for the compassion practices Develop and cultivate the qualities of the compassionate self Understanding the role of interpersonal difficulties and how stigma generates stress Cultivate compassion in my relationship with others Identify the main reasons people report fearing compassion Clarify what compassion is and what it is not Discuss the paradoxical effects of fears of compassion Help people to overcome their fears of compassion Cultivate self-compassion (of one’s body image, thoughts, and emotions) Build the capacity for acting compassionately toward one’s body Take the courage to engage in hard but necessary actions: The importance of physical activity	Compassion, loving-kindness, postures, facial expressions and voice tones, soothing rhythm breathing, compassionate imagery, compassion for others, fears of compassion, and self-compassion
Module 7: Final destination: A new start	Promote the early identification of relapse signals Distinguish lapse from relapse Draw an action plan to deal with the relapse Prevent new relapses	Relapse prevention, mindfulness, and committed action

In addition, the toolkit presented 2 other features, as follows:

1. Extra support, which was triggered when individuals reached a threshold of weight regain >3% above their target weight. This extra module stayed available until users returned to a weight interval of ±3% (ie, the interval defined as weight maintenance). For users in the *motivation and self-regulation* arm and the *emotion regulation* arm, this support further directed the user to content useful for coping with relapses (Multimedia Appendix 12).

2. Individualized feedback, which was included as a feature in the *motivation and self-regulation* arm and in which time series data retrieved over 1-2 months for each participant (activity and sleep patterns from Fitbit devices, daily and weekly body weight measures taken by the Fitbit Aria Wi-Fi scales, and use of the toolkit) were analyzed to examine weight change patterns. There were also indicators of weekly versus weekend and earlier versus later in the week used as possible predictors to alert participants to weekly cycles in weight change. Feedback could only be provided if sufficient data were collected (at least 30 days and variation in weight) and consisted of short messages such as “For you, being active or exercising seems linked to better weight management” or “Your weight management seems better on weekdays” that were displayed on the homepage.

#### Weekly Emails

During the intervention period, weekly emails were sent to the control arm, prompting the users to access the dashboard. These emails also included links to general information about weight management (eg, guidelines for healthy lifestyles from government or scientific association websites).

The other arms also received weekly emails prompting the users to access a specific session in the toolkit suggested for that week, that is, the participants from the *motivation and self-regulation* arm received the link for a session developed with that rationale, whereas the participants from the *emotion regulation* arm received the link to an emotion regulation–specific session.

### Step 4: Toolkit Technical Development

**Figure 6:**
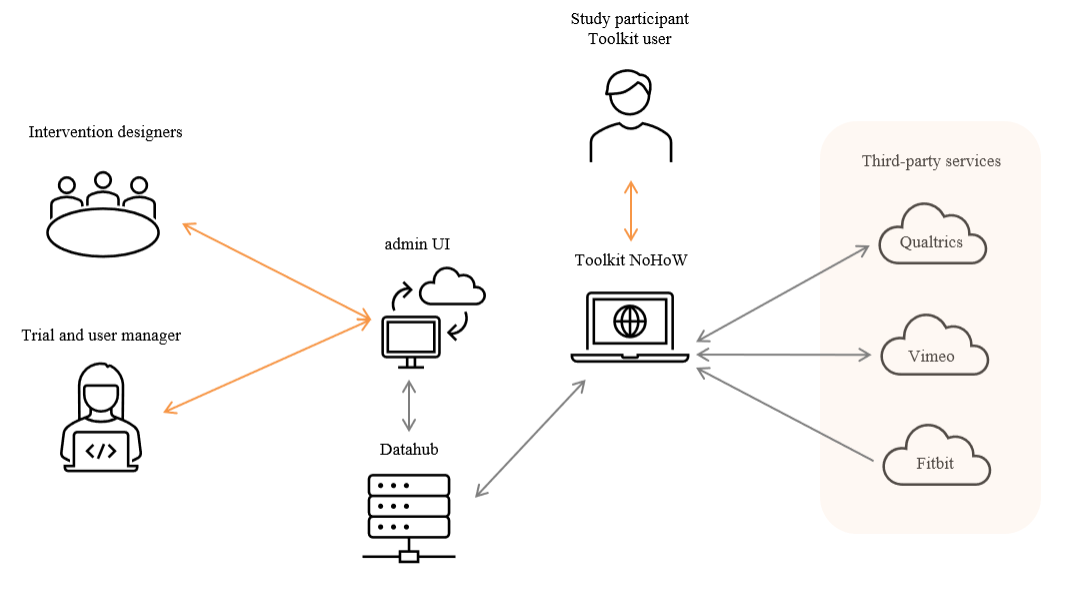
The overall architecture of the Navigating to a Healthy Weight Toolkit and its connections to third-party systems. NoHoW: Navigating to a Healthy Weight; UI: user interface.

Figure 6 depicts the final overall architecture of the NoHoW Toolkit and its connections to other systems required to deliver the intervention. The toolkit consisted of 2 layers. The front end consisted of an HTML skeleton with JavaScript modules. A responsive web design approach and mobile-first concept was used in the development. The back end responds to front end requests, communicates with the MySQL database on the datahub server, and accesses Fitbit data on Fitbit’s server. The datahub stores and analyzes data, providing personalized feedback to users by relaying information back to users through the personalized feedback panels on the dashboard of the toolkit. The intervention content was delivered through text, images, videos, questionnaires, and interactive exercises. The text and images were implemented as HTML. The videos were hosted on Vimeo (Vimeo, Inc) and embedded in the intervention sessions. The embedded questionnaires were implemented with Qualtrics (Qualtrics XM), which was also used for managing the study questionnaires in the RCT. New interfaces were built to retrieve questionnaire answers from Qualtrics and visualize them in the toolkit front end to enable exercises where instant feedback was needed. Interactive exercises were tailor made as mini apps. One example of a mini app was an exercise enabling the users to visualize key moments in their personal weight management history as a graph.

Each user was provided with a user account for the toolkit website as well as a Fitbit account to gather activity, sleep, and weight data. The personal map was designed iteratively with the help of a UX designer. First, 3 different concepts for visualizing the personal map were designed, and paper prototypes illustrating the concepts in different stages of use were created. The prototypes were evaluated with 6 participants in 2 evaluation rounds, first with 3 participants, after which major usability issues were fixed. Next, the updated prototypes were evaluated with another 3 participants. The evaluation consisted of 45-minute face-to-face sessions with each of the participants, who were asked to complete different tasks with the prototypes and provide feedback on the usability and visual appearance of the concepts. Finally, the participants were asked to rate the concepts as the best, second best, and worst. A concept illustrating a path on a map received the highest ratings and was selected. The final design was created by incorporating user feedback and some highly rated features from the other 2 concepts. All intervention modules were displayed on the main level of the map, and completed modules were highlighted in pink; modules that had not been completed were highlighted in a lighter shade of pink. When a user clicked on a module, the sessions of that module were displayed. This level of the map also indicated which of the sessions had been completed. By clicking on a session, the user gained access to the intervention content. On both levels of the map, there was also a box at the bottom left of the screen, indicating the next recommended session and providing a shortcut to the session.

The admin UI served intervention developers and trial managers. The most important component in terms of the intervention was the content management system (CMS) because of the large volume of intervention content and the need to constantly refine and update it during the iterative development of the toolkit. Thus, enabling the intervention designers to manage the content facilitated the collaboration between the software developers and the intervention designers. The CMS enabled the intervention designers to describe the intervention structure, content items, and their relationships to each other with a web-based UI without programming expertise. The CMS is based on the Django CMS (Django CMS Association), but its functionality has been tailored to meet the needs of the intervention design.

### Step 5: User Evaluation and Toolkit Refinement

A total of 37 eligible participants expressed interest in participating in the first study. Of the 37 participants, 20 (54%) started using the toolkit, 14 (38%) completed the 5-day questionnaire, and 9 (24%) completed the 30-day questionnaire. The average age of the participants was 41.4 (SD 11.7; range 23-63) years, and of the 37 participants, 22 (59%) were women. Although retention was low, the collected data were sufficient to allow for the analysis of the feasibility study. The results from the study were used to revise the toolkit specifications. The main threats to engagement and acceptance of the toolkit were identified, and several improvements were made regarding UX design, content development, technical performance, and usability (Multimedia Appendix 2). For example, to facilitate navigation in the toolkit, the personal map component was included, videos were refilmed to make them more energetic and less clinical, and technical performance and usability were improved by ensuring scaling of the toolkit to different screen sizes and ensuring compatibility with different browsers and mobile platforms. Ethical approval was obtained from the Psychology Research Ethics Committee of the University of Derby.

The qualitative study assessing users’ needs support when engaging with toolkit version 2.0 had a sample size of 12 adults aged 23-57 years (women: 9/12, 75%) who lost between 10% and 43% of their weight before taking part in the qualitative study. Most of the participants had experience in using digital tools, mainly to monitor their diet and physical activity. When testing the toolkit, the participants did not report difficulties, and the fact that the toolkit could be used on both a computer and mobile phone was considered an advantage. The participants considered that the self-monitoring tools were positive elements and considered that engaging in the toolkit sessions for 5-10 minutes per week was a reasonable time commitment. From the interviews, it was concluded that all participants felt that the toolkit content was acceptable; in particular, the whiteboard videos were considered to be very appealing and engaging in terms of both the design and content, with a participant stating, “I think I prefer the drawing hand, in my opinion the hand is more engaging, it has a lot of movement.” There were no major issues or suggestions for changes from the interviews (Multimedia Appendix 3). Approval from the research ethics committees at the University of Lisbon was obtained before recruitment and data collection.

The final version of the NoHoW Toolkit was presented in 4 versions available for each of the trial arms as well as in 3 languages for each of the trial centers. All versions included a tile-based dashboard displaying data from digital tracking devices (Fitbit Charge 2 wrist device and Aria smart scale) for weight, steps, and sleep, as well as manually entered data for eating and mood. It also included a diary section and a help section. In addition, the 3 versions available to each of the intervention arms—*motivation and self-regulation, emotion regulation*, and *combined*—displayed a personal map containing the modules of the toolkit. The final toolkit comprised 34 sessions, distributed through 15 modules, with active content being presented for 4 months. The *motivation and self-regulation* arm consisted of 17 sessions, distributed through 8 modules; the *emotion regulation* arm was presented with 17 sessions distributed through 7 modules; and the *combined* arm received the full toolkit. A detailed logging of user interactions with the toolkit was implemented to enable determining how often the users visited the toolkit and how much time they spent in the toolkit and in the intervention sessions.

## Discussion

### Principal Findings

The NoHoW Toolkit was one of the first theory and evidence-based digital approaches for WLM that was systematically developed using standardized guidance, an interdisciplinary approach, design principles, and user testing. In all, 4 toolkit versions were designed and formally evaluated through a 2×2 factorial RCT. In the RCT, the control version of the toolkit only presented self-monitoring information on weight and physical activities, whereas the 3 intervention arms of the toolkit presented additional modular content targeting (1) motivational factors of behavior change, including the promotion of autonomous motivation (vs controlled motivation), intrinsic goals, and flexible regulation to change behaviors and maintain weight loss and self-regulation skills; the setting and review of optimal goals; action and coping planning; and action control; (2) emotion regulation and stress management to reduce weight-related shame and self-criticism, reduce difficulties in emotion regulation, and increase psychological flexibility, mindfulness, and compassion; or (3) both 1 and 2. These are factors that have been shown to have an impact on energy balance–related behaviors (physical activity and healthy diet), weight changes, and well-being [12-15,35-38].

The systematic approach used in the development of the NoHoW Toolkit—making use of guidance for complex behavior change interventions and taxonomies—was a core contributor to the best reporting, replication, and accumulation of evidence on effective behavioral approaches to WLM. The logic models created for the NoHoW Toolkit allow for statistical modeling of the components of the intervention, contributing to understanding what works in the context of long-term weight management. Furthermore, using explicit theory when developing interventions allows for the identification of the factors that influence the target behavior, the mechanisms of actions that operate along the pathway to change, and the best techniques to influence these factors, as well as an understanding of how these can affect engagement. In addition, the use of digital tools and engagement metrics (eg, real-time data on weight or activity changes and use patterns of the intervention content) can contribute to improving our understanding of the behavioral dynamic patterns as well as theory testing and refinement. Users were engaged in the design and iteration of the toolkit at several stages, and the findings from these studies informed several improvements in the design of the toolkit and its content.

### Challenges and Lessons Learned

A challenge we faced in the development of the NoHoW Toolkit concerned the integration of the motivation and self-regulation theoretical approaches with emotion regulation to be tested in the 2×2 factorial trial. As there was no theory regarding, or empirical support for, the interactions between these approaches, the *combined* arm provided access to all the toolkit content available, and the users were prompted each week to access the emotion regulation content and the motivation and self-regulation content. In addition, there was some overlap among the sessions from the different approaches, for example, the sessions on values and on flexible eating. Furthermore, the content of the activities provided in the toolkit mainly focused on maintenance of weight loss by, for example, addressing the myths and facts of WLM, asking users to indicate the strategies they used to lose weight and how these strategies could fit with a long-term approach, and offering activities that addressed autonomous motivation as well as content that focused on mindfulness and compassion as ways to prevent relapses.

Although we used up-to-date frameworks to guide our choices for the content, structure, and flow of the toolkit, the final decisions on which components and features were to be included and how these would be implemented drew on the research team’s judgment through iterative discussions that were informed by user testing and feedback. It should be noted that the decisions made in the development process were not systemically justified and documented. Current frameworks and guidance provide little information on how to translate behavior change techniques into digital content (features). For example, what is the best way to implement a modeling technique in a digital intervention for it to be most effective? Should video or text or audio be used? Or should a combination of these be used? Behavior change techniques do not influence only the intervention content, but also the functionality of that content: how users interact with, and use, the app. This in turn affects how behavior change techniques can be translated into individual functionalities and how these functionalities form an overall concept that creates value for the user. Efforts are currently ongoing to develop tools to support researchers and interventionists in standardizing reporting to inform intervention design, content, and delivery [39,40].

The development of the NoHoW Toolkit involved close collaboration between behavioral scientists and web developers. The toolkit (and hence intervention) design was developed at the beginning of the project and limited by time and resource constraints. Where possible, development was accelerated by using existing commercial solutions, such as implementing intervention exercises using Qualtrics questionnaires and using commercially available tracking devices and their data aggregation capabilities. The resource constraints meant negotiating acceptable compromises between the teams in the implementation of some toolkit components. It was difficult to evaluate beforehand how much work each functionality required and how essential it actually was, considering the evidence base.

It can be challenging to develop a totally new digital behavior change intervention in a time-constrained project that includes a large-scale effectiveness evaluation. Although users were involved in the iterative development of the toolkit, it was not possible to simulate long-term, real-life experiences of users of such a large entity in qualitative interviews or even in a 1-month feasibility study. This compromise is also relevant when judging if the toolkit was suitable for participants from different socioeconomic status and literacy levels. Future studies should consider adopting methodologies such as coparticipatory design or formative research to improve the suitability of the app content for diverse populations. Another limitation related to this was that user testing was not conducted with Danish users.

The UX data collected during the NoHoW RCT will be valuable in understanding how users’ experiences change during the use of a long-term digital intervention. In future studies, when a complex long-term intervention is involved, it might be worthwhile to conduct a longer feasibility study to enable observation of the changes in user engagement and experience and use qualitative methods to investigate the reasons. Furthermore, an adaptive trial design might be a good alternative for testing and comparing different designs.

The schedule for the development was tight, which led to pressure to use existing commercial platforms in the development. Although including commercial options such as Fitbit can be an advantage because we can test real-world solutions, it also means less control over the features that are regularly added to the Fitbit app and trackers, which can contradict, or be in conflict or overlap with, the content provided in the NoHoW Toolkit, for example, the built-in *breathe* exercise in the Fitbit tracker was not aligned with the relaxation techniques recommended in the toolkit. Future interventions may favorably control these features by using devices used for research or codeveloping these with commercial partners, for example, by integrating new features on the Fitbit native app that would be released to the NoHoW participants only. Furthermore, developing a web app instead of a native mobile app limited the functionality and interaction mechanisms from the intervention design point of view. Focusing on the web-based app saved the trouble of developing separate apps for multiple platforms (Android and iOS), and the related CMS enabled updating of the intervention content in real time.

Although a factorial RCT allows a rigorous testing and comparison of different theory-based components, it also presents constraints regarding the continuous optimization of content to the individual that may arise during the trial (ie, the content could not be updated during the full length of the NoHoW trial). The use of optimization designs for digital interventions (eg, sequential multiple assignment randomized trial [41] or microrandomized trial [42] approaches) can support the identification and delivery of the content of interventions through the modeling of causal and time-varying effects, providing a more personalized and dynamic approach to complex behavior change in the context of long-term weight management.

Considering these challenges, it is likely that the interaction between academia and industry will be crucial to the development of digital behavior change solutions in the years to come. The industry has technical capabilities and capacity that could potentially synergize with academic efforts in developing rigorous, systematic, and dynamic behavior change interventions grounded in sound theoretical principles.

### Conclusions

The NoHoW Toolkit is a theory-based web app aimed at supporting individuals who have already lost weight and are trying to manage their weight in the long term. It was systematically developed using standardized guidance, an interdisciplinary approach, design principles, and user testing, and it was formally evaluated through a 2×2 factorial RCT. It presents modular content on motivational factors of behavior change as well as behavior regulation and emotion regulation skills, and it integrates data from activity trackers and weight scales. The development of the toolkit involved a multidisciplinary and international team of experts who used a systematic and rigorous approach to derive its technical specifications and content limitations. The toolkit has been tested in the context of a multicountry randomized trial; in addition to the main trial results, the results from the mediation-moderation analysis, guided by the logic models, will provide further information about the most effective and engaging components for WLM, thereby contributing to the optimization of the NoHoW Toolkit in future deployments.

## Appendix

Multimedia Appendix 1 Summary of a systematic review submitted as part of a deliverable in the Navigating to a Healthy Weight Project.

Multimedia Appendix 2 Summary of a feasibility study of the NoHoW Toolkit submitted as part of a deliverable in the NoHoW Project. NoHoW: Navigating to a Healthy Weight.

Multimedia Appendix 3 Summary of a qualitative analysis of the support needs and usability of the NoHoW Toolkit submitted as part of a deliverable in the NoHoW Project. NoHoW: Navigating to a Healthy Weight.

Multimedia Appendix 4 Theoretical guiding principles underlying the intervention design: motivation and behavior regulation arm.

Multimedia Appendix 5 Theoretical guiding principles underlying the intervention design: emotion regulation.

Multimedia Appendix 6 Graphical view of a tile: weight tile.

Multimedia Appendix 7 Personal route map.

Multimedia Appendix 8 Motivation and self-regulation modules.

Multimedia Appendix 9 Example of a whiteboard animation.

Multimedia Appendix 10 Example of a questionnaire.

Multimedia Appendix 11 Example of a testimony.

Multimedia Appendix 12 Extra support feature.

## Abbreviations

BCTT: Behavior Change Techniques Taxonomy
CMS: content management system
MBCT: Motivation and Behavior Change Techniques
NoHoW: Navigating to a Healthy Weight
RCT: randomized controlled trial
UI: user interface
UX: user experience
WLM: weight loss maintenance
