# Supplementary material for: A Theory- and Evidence-Based Digital Intervention Tool for Weight Loss Maintenance (NoHoW Toolkit): Systematic Development and Refinement Study
Source: J Med Internet Res. 2021 Dec 3;23(12):e25305. doi: 10.2196/25305 (PMC8686406; doi:10.2196/25305)
Supplement: Multimedia Appendix 1 [file jmir_v23i12e25305_app1.pdf]

Summary of a Systematic Review submitted as part of a deliverable in the NoHoW Project (Evidence-Based ICT Tools for Weight Loss Maintenance)

NoHoW has received funding from the European Union's Horizon 2020 research and innovation programme under grant agreement No 643309

Tittle: Systematic review of Behaviour change theories, techniques, and mode of delivery in digital-based lifestyle interventions aimed at weight management in adults.

Aims

1- Identify which theories of behaviour change have been used in digital-based interventions for weight management and how their use can be qualified. A major goal is to identify the extent to which theory-based mediators have been isolated and targeted by these interventions through the use of intervention techniques, and the quality with which this has been done.
2- Identify which theoretically-driven intervention techniques have been incorporated in digital-based interventions and how they have then been translated into MoDs.
3- Examine how characteristics of interventions - theoretical basis and extension of theory use, techniques, and MoDs - relate to effects on weight-related outcomes.

Methods

This systematic review and meta-analysis was conducted in accordance with the Preferred Reporting Items for Systematic Reviews and Meta-Analyses (PRISMA) statement (Liberati et al, 2009).

Eligibility criteria

Participants: Studies were included if they targeted overweight or obese adults (≥ 18 years old; BMI>24.9) or participants who had recently lost weight, regardless of weight status post weight loss. Studies involving children and adolescents, pregnant women, people with eating disorders, major depressive or anxiety disorders, as well as individuals participating in other interventions, including those involving surgical or pharmacological components, were excluded from the present systematic review. Chronic diseases, such as type 2 diabetes, hypertension or cardiovascular problems, were not used as exclusion criteria, because they represent common co-morbidities associated with overweight/obesity.

Interventions. This review was limited to behavioural interventions aimed exclusively or partially at managing weight, and that were delivered at least in part digitally. An intervention was considered ICT-based if at least one of the following delivery modes was included: networked-wireless tracking technologies; weighing scales and activity sensors; online tools; smartphone apps; multimedia resources; internet-based support, or if participants interacted directly with computer or mobile devices as part of the intervention. Interventions could present materials in several formats (i.e., text, sound, video, and animation).

Comparisons: With the exception of non-controlled trials, the comparators were arms consisting of a waiting-list control, usual care or other active interventions not comprising any digital components.

Outcomes: To be eligible, studies had to include data on at least one of the following outcomes – weight change, BMI, physical activity, or dietary behaviour, measured at baseline, post-treatment and/or follow-up.

Study design: Studies were included if they were intervention studies, using any type of experimental designs, including Randomised Controlled Trials, Non-randomised Controlled Trials and Non-Controlled Trials (with a pre-post intervention design). There were no restrictions with respect to the length of the intervention, assessment point(s), and setting.

<u>Search strategy and information Sources</u>

A comprehensive search of peer-reviewed articles in English published until April 2015 (including online ahead of print publication) was conducted in five electronic databases (Pubmed, PsycINFO, the Cochrane Library, Embase, and CINAHL). A comprehensive search strategy was used, with combinations of the following sets of terms: i) terms concerning the population of interest (overweight/obesity); ii) terms concerning the intervention(s) /exposure(s) evaluated (e.g., web-based interventions, mobile interventions); iii) terms in relation to the primary aim of the interventions (e.g., weight control, weight loss, weight maintenance); and iv) terms concerning the secondary outcomes of interest for NoHoW (e.g., dietary behaviour, physical activity, sedentary behaviour). An example search strategy is presented in Supplementary Material A.

<u>Study Selection</u>

Two researchers independently screened the titles and abstracts retrieved. Duplicate entries were handled electronically using a reference management software (Endnote® X7 for Mac® OS X®). Studies potentially meeting eligible criteria were then retrieved for a full review. The same researchers independently reviewed the full-text, and decisions to include or exclude studies in the review were made by consensus. A third author checked and approved the final selection of studies.

<u>Data extraction</u>

Data extraction for each study included information about: 1) bibliographic information (authors, year of publication, and reference); 2) sample characteristics (sample size, gender, age, BMI); 3) study design (randomised controlled trial, non-randomised controlled trial, non-controlled trial); 4) intervention characteristics (aim, format, length of intervention and follow-up); 5) Type of control group (when applicable); 6) Outcomes of interest; 7) drop-out rate.

One of two researchers independently extracted relevant information from each manuscript, which was checked by a second reviewer.

In addition, the following characteristics were coded: a) *Use of theory* - selected items of the Theory Coding Scheme (TCS) (Michie & Prestwick, 2010), which are relevant to intervention effectiveness, were used to code and evaluate *theory use* in the development of interventions. b) *Intervention techniques* - interventions were assessed for the presence of core *theoretically-driven techniques*, organised in: 1 - *self-regulation* theory principles (includes goal setting, planning, self-monitoring, feedback /progress evaluation, and relapse prevention), 2 - *self-determination theory* (includes autonomy, competence and relatedness-supportive techniques), and 3 - *emotional and stress regulation* (includes stress management, mindfulness, relaxation, managing negative or positive emotions). The name of the technique(s) used was recorded. c) *Modes of delivery* (MoDs) of interventions were coded using the scheme developed by Webb and colleagues (2010).

The presence or absence of these characteristics was coded for each eligible experimental trial arm. Two researchers independently coded this information. A third researcher resolved discrepancies.

<u>Methodological Quality</u>

Study quality was assessed using the Quality Assessment Tool for Quantitative Studies, developed by the Effective Public Health Practice Project, and recommended for use by the Cochrane Public Health Review Group (Higgins & Green, 2011). This tool evaluates six key methodological domains: study design, blinding, representativeness (selection bias), representativeness (withdrawals/dropouts),

confounders, and data collection. Each domain is classified as *Strong, Moderate* and *Weak* methodological quality based on specific criteria. A global rating is calculated based on the scores for each component. Two researchers independently rated each of the six domains and overall quality. A third researcher resolved discrepancies.

Data synthesis

Results were described in a narrative summary. Study characteristics and data related to the use of theory, intervention techniques and MoDs were aggregated to report frequencies. In addition, for studies presenting significant differences between arms, we examined the relation between intervention effects and use of theory, intervention techniques and MoDs. Effect sizes (ES) were therefore extracted for each of the trials presenting significant differences between arms. If this information was unavailable in the published study, ES were calculated based on the statistical information available (e.g. sample sizes and means and standard deviations, mean change, SD difference), using an appropriate software (Comprehensive Meta-Analysis software version 2; Borenstein, 2005). Effect sizes were the standardized mean difference (*d*), interpreted according to Cohen's guidelines (1992) (values of 0.20, 0.50 and 0.80 correspond to small, medium and large effect sizes). If there was more than one follow-up assessment point available, the longest period available was chosen. In the case of studies presenting two or more intervention arms meeting eligibility criteria, the arm representing the ICT intervention with the most intensive behavioural components was chosen. Intervention arms were compared against control groups, and in the case of non-controlled trials, compared against the most passive comparison condition. Data from intention-to-treat analyses were used whenever reported in primary studies.

Results

Study selection

The literature search yielded a total of 267 potentially relevant records. After exclusion of 99 duplicates, 168 abstracts were assessed for eligibility. After the screening of titles and abstracts 96 studies were excluded. Assessment of the full-text of 72 studies further excluded 27 articles. Reasons for exclusion were the study design, target population, type of treatment provided, type of article, and outcomes. Forty-five studies reporting 34 unique trials interventions that met eligibility criteria were included in this systematic review (Figure 1).

Methodological Quality

Results for methodological quality assessment are available in Table 1 (for detailed classifications of each item see Supplementary Material B). Overall, nineteen trials were rated as having *moderate* overall methodological quality, 12 were evaluated as *weak,* and 3 trials presented *strong* methodological quality. For Selection Bias criteria the majority of trials (k=24) were scored as *weak* (targeted population self-referred from specific source), indicating that study samples may not represent the target population. Twenty-five studies were scored as *strong* on Controlling for Confounders. For the remaining categories, most trials were rated as of moderate quality.

Study and participants' characteristics

Characteristics of included studies are presented in Table 1. In total, 14806 participants were included in this systematic review (individual study range: n=47 to n=2862), with a mean age of 48 years, and 72% were women (5 studies targeted women only). Initial BMI ranged between 26.1 and 35.6 (mean=28). Most reported interventions were RCTs (k=28), only 1 was a NCT, most targeted weight management (k=14) and weight loss (k=14). Only 4 trials targeted weight loss maintenance. Interventions lasted from 6 to 144 months (mean = 40.5). Six trials included a follow-up assessment

period, from 12 to 96 weeks. Attrition ranged from 3.4% to 60.2% (mean=21.6%). The most common primary outcomes were weight change (k=27), BMI change (k=8), physical activity (k=8) and dietary intake (k=4).

Theoretical basis and use of Theory

Half of the trials (k=17) were at least informed by one or more theories of behaviour change. Of these, 11 (65%) adopted a single theoretical framework, while 6 (35%) used a combination of theories. The most frequent theory was Social Cognitive Theory (SCT), either isolated (k=6), or in combination with the Transtheoretical Model (TTM; k=3). Two trials adopted a Self-Determination Theory framework and 2 others adopted the TTM. The remaining 4 trials reported using a combination of various other theories. Twenty studies (59%) mentioned a psychological (target) construct as a predictor of behaviour, and 16 (47%) used predictors or theory to select and develop intervention techniques (Table 2; Full details present in Supplementary Material C).

Intervention techniques

Thirty-one trials reported using at least one technique to change behaviour and/or manage weight. Self-regulation based techniques were the most commonly used (k= 30, 86%), namely goal setting, planning, self-monitoring and provision of feedback. Sixteen of these trials provided full details of how techniques were applied, and 13 mentioned using them but without providing details or examples. Next, 9 trials (26%) reported using motivational techniques, such as motivational interviewing and providing choice, but only 1 trial provided full details of how these techniques were implemented. Finally, 4 trials (12%) used emotional regulation techniques, mainly stress management, all of which provided insufficient detail on its use. (Table 3; Full details on the techniques used are present in Supplementary Material C).

Modes of delivery

Almost all trials reported using Automated functions (k=33), mainly *Automated Tailored feedback* based on progress monitoring such as reinforcing messaging (k=23, 45%) and *Enriched Information Environment* such as testimonials or videos (k=18, 35%). Sixteen trials used supplementary MoDs, of which email was the most frequent (k=13, 54%). Regarding Communicative functions (k=15), providing peer-to-peer access (e.g. buddy systems) and access to an advisor to request advice (e.g. expert-led discussion board) were the most frequently reported (k=7, 47%, and k=5, 43%, respectively). (Table 4; Full details of the MoDs are present in Supplementary Material C).

Relation between treatment effects and use of theory, intervention techniques and modes of delivery

Table 5 presents the effect sizes (ES) of studies reporting significant differences between arms for the primary outcome of each study. Thirteen trials (39%) reported significant differences between arms. Of these, eleven favoured computerised intervention, two favoured a behaviour change enhanced internet intervention vs. a basic educational Internet intervention, one did not favour the interactive technology-based intervention vs. self-directed control, and another favoured the non-computerised intervention (manual-based intervention). With the exception of the study by Roesch and colleagues (2010) that reported ES for physical activity, all other studies reported ES for weight changes. Effect sizes varied between d=-0.65 (95% CI -1.24-0.06) and d=1.00 (95% CI 0.51- 1.49).

The three studies presenting larger effects, reported using both self-regulation (goal setting, self-monitoring and feedback) and stress management techniques. On the other hand, two studies reporting trivial effects and the study by Womble et al, which presented an ES favouring the control group, did not use any techniques derived from the theoretical frameworks considered in the present review. Self-regulation techniques were present in all studies reporting significant effect sizes. Only five of these trials used a theoretical framework to inform the intervention, and theories selected varied between trials and do not seem to be associated with ES. All interventions used

Automated Functions, and only five trials used only one MoD. MoDs employed do not seem to be associated with the ES of studies.

Reviewing the content of the three interventions that showed medium to large ES, we found that they all included an educational component in weight management strategies. In addition, participants in these interventions were instructed to set goals and self-monitor their dietary intake and physical activity (and weight in Adachi's intervention (2007) using tools provided in the trial, e.g. computer software programs which enabled submission of self-monitoring records to the study website. In all three interventions, participants received automated tailored feedback on their behaviour and progress, based on their uploaded records. The three interventions with the largest ES also included an emotional regulation component (stress management), although this was only an educational component on stress management instead of stress management training. Additional behavioural strategies (e.g. problem solving, stimulus control) were included in all three interventions.

References

Cohen, J. (1992). A power primer. *Psychological Bulletin*, 112(155-9).

Higgins, J., Green, S. eds. (2011). *Cochrane Handbook for Systematic Reviews of Interventions Version 5.1.0*. The Cochrane Collaboration.

Michie, S. Prestwich, A. (2010). Are interventions theory-based? Development of a theory coding scheme*. Health Psychology*, 29(1): 1-8. doi: 10.1037/a0016939.

Webb, T. Joseph, J., Yardley, L., Michie, S. (2010). *Using the internet to promote health behavior change: A systematic review and meta-analysis of the impact of theoretical basis, use of behavior change techniques, and mode of delivery on efficacy. Journal of Medical Internet Research,* 12(1): e4. doi: 10.2196/jmir.1376.

Figure 1 Flow Diagram of studies

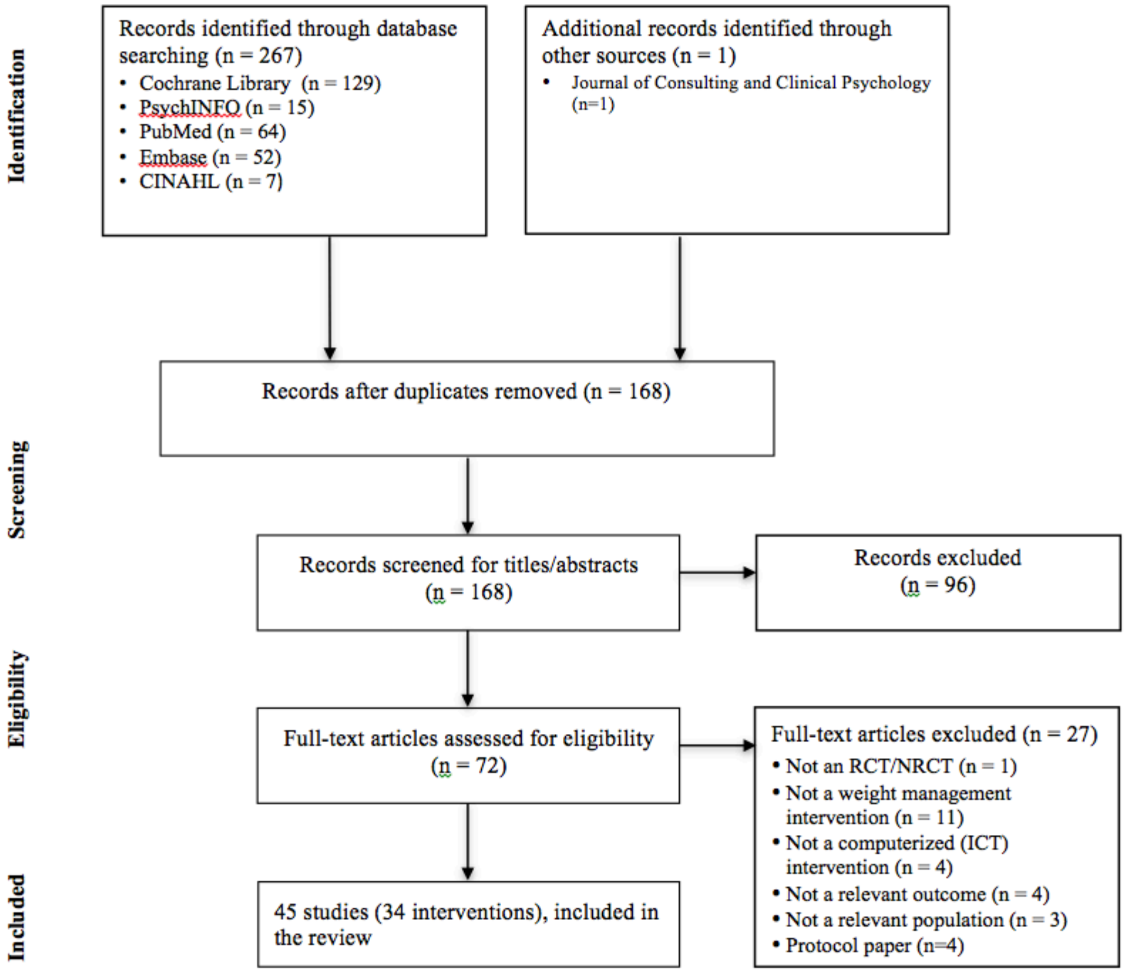

Tables

Table 1 Characteristics of included studies

Study ID	Study design	Sample			Intervention		Attrition (%)	Outcomes of interest	Quality assessment
		Size/ % women	Age (years)*	BMI (kg/m^2)*	Aim	Length + follow-up assessment (weeks)			
**Adachi, 2007**	RCT; 4 arms : 1) Computerized program + self-monitoring 2) Computerized program 3) Booklet + self-monitoring 4) Booklet	205/100	46.2 ± 9.5	26.1 ± 1.5	Weight management	28	3.4	*Primary:* weight, BMI *Secondary:* DI, PA, steps	Moderate
**Anderson-Bill, 2011**	NCT; 2 arms: a) Web-based Guide to Health (GTH); and b) GTH enhanced.	272/86	43.7 ± 10.4	29.2 ± 3.8	Improve PA, nutrition and weight management	64		*Primary:* walking *Secondary:* weight, DI	Weak
**Burke, 2009, 2012 Ambeba, 2015**	RCT; 3 arms: 1) Standard paper record 2) Personal digital assistant 3) Personal digital assistant + feedback	210/85	49.0 ± 13.0	33.1 ± 6.9	Weight loss and maintenance	96	24.0	*Primary:* weight	Moderate
**Carlson, 2012**	2 RCTs; 2 arms each: 1) Web-based program 2) Wait-list control and 1) Web-based program 2) Generic information website	352/52	43.3 ± 8.3	32.8 ± 4.4	Weight loss	48	29.9	*Primary:* DI, PA, sedentary time	Weak
**Carter, 2013**	RCT; 3 arms: 1) Smartphone app intervention 2) Website intervention 3) Paper diary intervention	128/77	41.9 ± 9.1	34.2 ± 5.2	Weight management	24	-	*Secondary:* weight, BMI	Weak
**Castelnuovo, 2010, 2011**	RCT; 2 arms: 1) Computerized intervention 2) Usual care	72/-	53.3 ± 8.4	≥30	Weight loss	52	-	*Primary:* weight	Weak

Study ID	Study design	Sample			Intervention		Attrition (%)	Outcomes of interest	Quality assessment
		Size/ % women	Age (years)*	BMI (kg/m²)*	Aim	Length + follow-up assessment (weeks)			
**Chambliss, 2011**	RCT; 3 arms: 1) Computerized self-monitoring + basic feedback 2) Computerized self-monitoring + enhanced behavioural feedback 3) Wait-list control	120/83	45.0 ± 10.3	30.5 ± 2.6	Weight management	12	21.1	*Primary:* weight, BMI	Moderate
**Chung, 2014**	RCT; 3 arms: 1) Food diary 2) Electronic diary 3) Assessment –only control	60/63	37.4 ± 11.2	27.7 ± 3.1	Weight loss	12	10.0	*Primary:* weight, BMI	Weak
**Cussler, 2008**	RCT; 2 arms: 1) Internet contact 2) Self-directed	135/100	48.0 ± 4.4	30.7 ± 3.6	Weight loss maintenance	48	17.8	*Primary:* weight, BMI, EI *Secondary:* internet use	Moderate
**Genugten, 2012**	RCT; 2 arms 1) Tailored intervention 2) Generic information website	539/69	47.8 ± 9.4	28.0 ± 1.9	Weight management	8 + 24	34.8	*Primary:* BMI, PA, fat intake, snacks and sweetened drinks	Moderate
**Haapala, 2009**	RCT; 2 arms: 1) Mobile phone intervention 2) Control	125/78	38.1 ± 4.7	30.5 ± 2.8	Weight loss	48	32.0	*Primary:* weight, DI, PA	Moderate
**Hersey, 2012**	RCT; 3 arms: 1) Basic web access 2) Basic web access + interactive website 3) Basic web access + interactive website + telephone coaching support	1755/74	46.7 ±	≈33.6	Weight loss	48 + 60-72	30.8	*Primary:* weight	Weak
**Johnson, 2008**	RCT; 2 arms: 1) Home-based stage-matched multiple	1277/47	45.4 ±	≈30.8	Weight management	48 + 96	60.2	*Primary:* healthy eating, PA	Moderate

Study ID	Study design	Sample			Intervention		Attrition (%)	Outcomes of interest	Quality assessment
		Size/ % women	Age (years)*	BMI (kg/m²)*	Aim	Length + follow-up assessment (weeks)			
	behaviour intervention 2) Assessment-only control							*Secondary:* weight	
**LaChausse, 2012**	RCT; 3 arms: 1) Online course 2) On-campus course 3) Comparison group	358/66	24.9 ± 8.4	29.0 ± 7.9	Weight management	12		*Primary:* BMI, dietary and PA behaviours	Weak
**Kirk, 2003 McConnon, 2007**	RCT; 2 arms: 1) Internet intervention 2) Usual care	221/77	45.8 ± 10.6	≈34.4 (median)	Weight management	48	41.0	*Primary:* weight, BMI	Weak
**Morgan, 2010, 2014 Collins, 2011**	RCT; 3 arms: 1) Resources intervention 2) Online intervention 3) Wait-list control	159/0	18-65	25-40	Weight loss	12+12	19.6	*Primary:* weight *Secondary:* PA, DI, sedentary behaviour	Strong
**O'Brien, 2014**	Prospective RCT; 3 arms: 1) Standard online intervention 2) Enhanced online intervention 3) Wait-list control	289/60	41.6 ± 10.2	32.3 ± 3.9	Weight loss	12		*Primary:* weight, BMI *Secondary:* DI, eating behaviours, PA	Weak
**Patrick, 2009**	RCT (Pilot Study); 2 arms: 1) Web-based intervention 2) Usual care	65/80	44.9 ± 7.7	33.2 ± 4.4	Weight loss	16	17.0	*Primary:* weight	Weak
**Pellegrini, 2012**	RCT; 3 arms: 1) Standard intervention 2) Standard intervention + technology-based system 3) Technology-based system	51/86	44.2 ± 8.7	33.7 ± 3.6	Weight loss	24	23.5	*Primary:* weight Secondary: BMI, PA, DI	Weak
**Quintiliani, 2014**	RCT; 2 arms: 1) Intervention 2) Assessment-only control	211/100	38.1 ± 7.6	31.1 ± 7.7	Improve weight, diet and PA	144	-	*Primary:* weight *Secondary:* DI, PA	Moderate

Study ID	Study design	Sample			Intervention		Attrition (%)	Outcomes of interest	Quality assessment
		Size/ % women	Age (years)*	BMI (kg/m^2)*	Aim	Length + follow-up assessment (weeks)			
**Roesch, 2010**	RCT; 2 arms: 1) Web-based intervention 2) Assessment-only control	842/48	42.6 ± 8.4	≥25	Improve PA and diet for weight loss	52		*Primary:* PA	Moderate
**Rothert, 2006**	RCT; 2 arms: 1) Web-based tailored expert system 2) Web-based information-only intervention	2862/83	45.4 ± 12.1	32.1 ± 3.9	Weight management	6 + 24		*Primary:* weight	Moderate
**Shapiro, 2012**	RCT; 2 arms: 1) Intervention 2) Control (minimal intervention contact)	170/65	41.9 ± 11.8	32.2 ± 4.1	Weight loss	52	24.0	*Primary:* weight *Secondary:* steps	Moderate
**Brantley, 2008 Stevens, 2008 Svetkey, 2008, 2014**	RCT; 3 arms: 1) Personal contact intervention 2) Interactive technology-based intervention 3) Self-directed control	1032/63	56.0 ± 9.0	34.1 ± 4.8	Weight loss maintenance	128	6.6	*Primary:* weight *Secondary:* EI, PA	Strong
**Tate, 2001**	NRCT; 2 arms: 1) Internet education 2) Internet behaviour therapy	91/89	40.9 ± 10.6	29.0 ± 3.0	Weight loss	24	22.0	*Primary:* weight *Secondary:* DI, PA	Moderate
**Tate, 2003**	NRCT; 2 arms: 1) Web-based intervention 2) Web-based + behavioural e-counseling intervention	92/89	48.5 ± 9.4	33.1 ± 3.8	Weight loss	52	16.3	*Primary:* weight *Secondary:* DI, PA	Moderate

Study ID	Study design	Sample			Intervention		Attrition (%)	Outcomes of interest	Quality assessment
		Size/ % women	Age (years)*	BMI (kg/m²)*	Aim	Length + follow-up assessment (weeks)			
**Thorndike, 2012**	RCT; 2 arms: 1) Web-based and personal contact intervention 2) Usual care	330/86	42.9 ± 12.7	≥25	Weight loss maintenance	38	21.5	*Primary:* weight, PA, DI	Moderate
**Ware, 2008**	NCT	233/51	40.9 ± 8.1	27.1 ± 4.8	Improve PA and weight management	12		*Primary:* weight, PA	Moderate
**Webber, 2010a, 2010b**	NRCT; 2 arms: 1) Standard intervention 2) Motivation-enhanced intervention	66/100	50.1 ± 9.9	31.1 ± 3.7	Weight loss	16	12.0	*Primary:* weight, self-monitoring	Moderate
**Webber, 2013**	NRCT; 2 arms: 1) Web-based intervention 2) Web-based intervention + portion-controlled diet	50/86	46.0 ± 10.7	35.1 ± 3.8	Weight loss	12	6.0	*Primary:* weight	Moderate
**Werkman, 2010**	RCT; 2 arms: 1) Intervention 2) Control (minimal intervention contact)	413/15	59.5 ± 2.4	27.0 ± 3.4	Weight gain prevention	52 + 52	16.0	*Primary:* weight, DI, PA	Weak
**Wier, 2009**	RCT; 3 arms: 1) Usual care + phone counseling 2) Usual care + web-based intervention + e-mail counseling 3) Usual care	1386/33	43.0 ± 8.6	29.6 ± 3.5	Weight management	24	18.5	*Primary:* weight *Secondary:* PA, DI	Strong
**Womble, 2004**	RCT; 2 arms: 1) Web-based intervention 2) Manual	47/100	43.7 ± 10.2	33.5 ± 3.1	Improve weight	52	34.0	*Primary:* weight *Secondary:* eating behaviour	Moderate
**Wylie-Rosett, 2001**	RCT; 3 arms: 1) Workbook 2) Workbook + computerized tailoring 3) Workbook +	588/82	52.2 ± 12.3	35.6 ± 6.5	Weight loss	52	18.3	*Primary:* weight *Secondary:* DI, PA	Moderate

Study ID	Study design	Sample			Intervention		Attrition (%)	Outcomes of interest	Quality assessment
		Size/ % women	Age (years)*	BMI (kg/m^2)*	Aim	Length + follow-up assessment (weeks)			
	computerized tailoring + staff consultation								

Note: * Mean ± Standard Deviation; NRCT: non-randomized controlled trial; RCT: randomized controlled trial; NCT – Non-controlled trial; BMI: body mass index; DI: dietary intake; EI: energy intake; PA: physical activity

Table 2 Theoretical Basis and Use of Theory in included studies

Use of Theory	No		Yes	
	**N**	**%**	**N**	**%**
Theoretical basis	17	50	17	50
Single Theory	6	35	11	65
Target construct related behaviour	14	41	20	59
Theory used to select technique	18	53	16	47
Name of Theory	**N**		**%**	
SCT	6		35	
TTM	2		12	
SDT	2		12	
Model of Community Nutrition Environments	1		6	
SCT + TTM	3		18	
SDT, TPB, PAPM	1		6	
Systems Contingency Model; SET	1		6	
Social Comparison, DBT	1		6	

Note: SCT: Social-Cognitive Theory; SDT: Self-Determination Theory; SET: Self-Efficacy Theory; TPB: Theory of Planned Behaviour; TTM: Transtheoretical Model; PAPM: Precaution Adoption Process Model; DBT: Dialectical Behavior Theory.

Table 3 Techniques employed in included studies

Intervention techniques	Yes	
	N	%
Self-Regulation		
0	5	14
1	14	40
2	16	46
Motivation		
0	25	71
1	9	26
2	1	3
Emotion Regulation		
0	30	88
1	4	12
2	0	0

Note: Frequencies correspond to techniques reported for each digital experimental arms of the studies included.
0: Not mentioned; 1: Mentioned explicitly, but unspecified; 2: Techniques used mentioned explicitly and specified how.

Table 4 Modes of Delivery used in Included Studies

Modes of Delivery	Yes	
	N	%
Automated Functions		
a. Enrichment information environment	18	35
b. Automated tailored feedback	23	45
c. Automated follow-up messages	10	20
Communicative Functions		
d. Access to advisor to request advice	5	33
e. Scheduled contact with advisor	3	20
f. Peer-to-peer access	7	47
Additional Modes		
g. Email	13	54
h. Telephone	5	21
i. Text message (SMS)	2	8
j. CD-ROM	4	17

Table 5 - Studies with differences between groups ordered by effect size, and theoretical basis, intervention techniques and modes of delivery used

Study ID	Effect Sizes	Theoretical basis	Intervention techniques			Mode of Delivery		
			Self-Regulation	Motivation	Stress/emotional regulation	Automated Functions	Communicative Functions	Additional Modes
Chambliss, 2011	1.00 (0.51-1.49)		X		X	X		X
Tate, 2001	0.68 (0.20-1.16)		X		X	X	X	X
Adachi, 2007	0.59 (0.16-1.03)		X		X	X		
Tate, 2003	0.40 (-0.01-0.82)		X			X	X	X
Wylie-Rosett, 2001	0.29 (0.22-0.35)	TTM	X			X		
Rothert, 2006	0.29 (0.13-0.45)		X			X		X
Patrick, 2009	0.28 (-0.26-0.83)		X			X	X	X
Haapala, 2009	0.26 (-0.18-0.69)	Systems Contingency Model; SET	X			X		
Roesch, 2010	0.21 (0.07-0.34)	SCT, TTM	X			X	X	X
Johnson, 2008	0.17 (0.01-0.33)	TTM				X		
Carter, 2013	0.10 (-0.33-0.52)					X		
Svetkey, 2008	-0.04 (-0.19-0.11)	SCT, TTM		X	X		X	X
Womble, 2004	-0.65 (-1.24—0.06)					X		X

Note: SCT: Social-Cognitive Theory; SDT: Self-Determination Theory; SET: Self-Efficacy Theory; TPB: Theory of Planned Behaviour; TTM: Transtheoretical Model.

Supplementary Materials

Supplementary Material A

Example of the search strategy (PsycInfo)

S1	(web-based OR internet OR digital OR online OR technolog* OR computer OR mobile) AND behavio* change AND (interventions OR programs)	1,514
S2	weight control OR weight loss OR weight maintenance OR weight management OR obesity treatment	15,799
S3	random* controlled trial OR random* OR random* trial OR clinical trial	155,858
S4	nutrition OR diet OR dietary OR "physical activity" OR exercise OR sedentary behav*	105,724
S5	overweight OR obes*	31,062
S6	S1 AND S2 AND S3 AND S4 AND S5	15

Supplementary Material B

Table B.1. Quality assessment of included studies

Study	Selection Bias	Study Design	Confounders	Blinding	Data Collection	Withdrawals	Final Rating
Adachi, 2007	weak	strong	strong	moderate	moderate	strong	moderate
Anderson-Bill, 2011	weak	moderate	weak	moderate	strong	weak	weak
Burke, 2009	moderate	strong	strong	moderate	weak	moderate	moderate
Carlson, 2012	weak	strong	weak	moderate	weak	weak	weak
Carter, 2013	weak	strong	strong	moderate	strong	weak	weak
Castelnuovo, 2011	weak	strong	strong	moderate	strong	weak	weak
Chambliss, 2011	weak	strong	strong	moderate	strong	moderate	moderate
Chung, 2014	weak	strong	strong	strong	strong	weak	weak
Cussler, 2008	weak	strong	strong	moderate	moderate	strong	moderate
Genugten, 2012	weak	strong	strong	moderate	moderate	strong	moderate
Haapala, 2009	weak	strong	strong	moderate	moderate	moderate	moderate
Hersey, 2012	moderate	strong	strong	weak	moderate	weak	weak
Johnson, 2008	moderate	strong	weak	moderate	strong	moderate	moderate
LaChausse, 2012	weak	strong	weak	weak	strong	strong	weak
McConnon, 2007	weak	strong	strong	weak	strong	weak	weak
Morgan, 2010	moderate	strong	weak	strong	moderate	strong	strong
O'Brien, 2014	weak	strong	strong	moderate	moderate	weak	weak
Patrick, 2009	weak	strong	strong	weak	strong	strong	weak
Pellegrini, 2012	weak	strong	weak	moderate	strong	moderate	weak
Quintilliani, 2014	strong	strong	strong	moderate	strong	weak	moderate
Roesch, 2010	moderate	strong	strong	moderate	moderate	weak	moderate
Rothert, 2006	moderate	strong	strong	moderate	strong	weak	moderate

Table B.1. (cont.) Quality assessment of included studies

Study	Selection Bias	Study Design	Confounders	Blinding	Data Collection	Withdrawals	Final Rating
Shapiro, 2012	weak	strong	strong	moderate	moderate	moderate	moderate
Svetkey, 2008	moderate	strong	strong	moderate	strong	moderate	strong
Tate, 2001	weak	strong	strong	moderate	moderate	moderate	moderate
Tate, 2003	weak	strong	strong	moderate	moderate	strong	moderate
Thorndike, 2012	weak	strong	strong	moderate	moderate	moderate	moderate
Ware, 2008	weak	moderate	strong	moderate	moderate	moderate	moderate
Webber, 2010	weak	strong	strong	moderate	strong	strong	moderate
Webber, 2013	weak	strong	strong	moderate	moderate	strong	moderate
Werkman, 2010	weak	strong	moderate	weak	moderate	strong	weak
Wier, 2009	moderate	strong	strong	moderate	moderate	moderate	strong
Womble, 2004	weak	strong	moderate	moderate	moderate	moderate	moderate
Wyley-Rosett, 2001	moderate	strong	moderate	moderate	weak	moderate	moderate

Supplementary Material C

Table C.1. Theoretical basis and use of theory, intervention techniques and mode of delivery of included studies

**Study ID**	**Use of theory**				**Techniques**			**Mode of delivery**		
	Theoretical basis	Single Theory	Target constr. related behaviour	Theory used to select technique	Self-Regulation	Motivation	Emotional regulation	Automated Functions	Communicative Functions	Additional Modes
Adachi, 2007	No	-	No	No	1	0	1	b. Automated tailored feedback		
Anderson-Bill, 2011	SCT	Yes	Yes	Yes	Both groups: 2	Basic: 1<br>Enhanced: 2	0	b. Automated tailored feedback		
Burke, 2009<br>Burke, 2012<br>Ambeba, 2015	SCT	Yes	Yes	Yes	Both groups: 2	1	0	b. Automated tailored feedback		
Carlson, 2012	SCT, TTM	No	No	No	2	0	0	b. Automated tailored feedback		
Carter, 2013	No	-	Yes	No	2	0	0	a. Enrichment information environment<br>b. Automated tailored feedback		
Castelnuovo, 2011<br>Castelnuovo, 2010	SDT	Yes	Yes	Yes	2	1	1	a. Enrichment information environment		
Chambliss, 2011	No		Yes	No	Both groups: 2	0	1	b. Automated tailored feedback		g. Email<br>h. Telephone
Chung, 2014	No	-	Yes	Yes	1	0	0	b. Automated tailored feedback		
Cussler, 2008	No	-	No	No	1	0	0	a. Enrichment information environment<br>b. Automated tailored feedback	d. Access to advisor to request advice	

Genugten, 2012	SDT, TPB, PAPM	No	Yes	Yes	0	0	0	a. Enrichment information environment		
Haapala, 2009	Systems Contingency Model; SET	No	Yes	No	1	0	0	a. Enrichment information environment<br>b. Automated tailored feedback		
Hersey, 2012	No	-	No	No	1	1	0	a. Enrichment information environment<br>b. Automated tailored feedback		h. Telephone
Johnson, 2008	TTM	Yes	Yes	Yes	0	0	0	b. Automated tailored feedback		
McConnon, 2007	No	-	Yes	No	2	0	0	a. Enrichment information environment<br>c. Automated follow-up messages		g. Email
LaChausse, 2012	No	-	No	No	1	0	0	a. Enrichment information environment<br>b. Automated tailored feedback		
Morgan, 2010<br>Morgan, 2014<br>Collin, 2011	SCT	Yes	Yes	Yes	2	0	0	a. Enrichment information environment<br>b. Automated tailored feedback		j. CD-ROM
O'Brien, 2014	SCT	Yes	Yes	Yes	2	1	0	a. Automated tailored feedback<br>b. Automated follow-up messages	f. Peer-to-peer access	g. Email<br>h. Telephone<br>i. Text message (SMS)
Patrick, 2009	No	-	Yes	Yes	2	0	0	b. Automated tailored feedback	d. Access to advisor to request advice	h. Telephone

Pellegrini, 2012	No	-	No	No	0	0	0	b. Automated tailored feedback	e. Scheduled contact with advisor	
Quintiliani, 2014	Model of Community Nutrition Environments	Yes	Yes	Yes	0	0	0		f. Peer-to-peer access	j. CD-ROM
Roesch, 2010	SCT, TTM	No	Yes	Yes	1	0	0	a. Enrichment information environment	e. Scheduled contact with advisor	g. Email<br>h. Telephone
Rothert, 2006	No	-	No	No	1	0	0	b. Automated tailored feedback<br>c. Automated follow-up messages		g. Email
Shapiro, 2012	SCT	Yes	Yes	No	2	0	0	a. Enrichment information environment<br>b. Automated tailored feedback<br>c. Automated follow-up messages		g. Email
Svetkey, 2008<br>Stevens, 2008<br>Brantley, 2008<br>Svetkey, 2014	SCT, TTM	No	Yes	Yes	2	1	0	a. Automated tailored feedback<br>b. Automated follow-up messages	d. Access to advisor to request advice	g. Email<br>j. CD-ROM
Tate, 2001	No	-	No	No	1	0	1	a. Automated tailored feedback	d. Access to advisor to request advice<br>f. Peer-to-peer access	g. Email

Tate, 2003	No	-	No	No	Web-based: 1<br>Web-based + e-counseling: 2	0	0	a. Enrichment information environment<br>c. Automated follow-up messages	e. Scheduled contact with advisor	g. Email
Thorndike, 2012	No	-	No	No	2	0	0	b. Automated tailored feedback	d. Access to advisor to request advice	g. Email
Ware, 2008	Social Comparison, DBT	No	Yes	Yes	2	0	0	a. Enrichment information environment<br>b. Automated tailored feedback<br>c. Automated follow-up messages	f. Peer-to-peer access	g. Email<br>i. Text message (SMS)
Webber, 2010a<br>Webber, 2010b	SDT	Yes	Yes	Yes	1	1	0	a. Enrichment information environment	f. Peer-to-peer access	
Webber, 2013	No	-	No	No	1	0	0	a. Enrichment information environment	f. Peer-to-peer access	
Werkman, 2010	No	-	Yes	Yes	2	1	0	a. Enrichment information environment<br>b. Automated tailored feedback<br>c. Automated follow-up messages	f. Peer-to-peer access	g. Email<br>j. CD-ROM
Wier, 2009	SCT	Yes	No	Yes	1	0	0	a. Enrichment information environment<br>c. Automated follow-up messages		
Womble, 2004	No	-	No	No	0	0	0	c. Automated follow-up messages		g. Email
Wylie-Rosett, 2001	TTM	Yes	No	No	Both groups: 1	1	0	a. Enrichment information environment		

Supplementary Material D

Table D.1. Full details of techniques used in included interventions

	Techniques			
**Study**	Self-Regulation	Motivation	Stress/Emotional Regulation	Other techniques
**Adachi, 2007**	- Goal setting<br>- Self-monitoring<br>- Feedback		- Stress management	- Social support<br>- Pros and cons<br>- Stimulus control<br>- Cognitive restructuring
**Anderson-Bill, 2001**	- Goal setting<br>- Planning<br>- Self-monitoring<br>- Feedback<br>- Goal revision	- Choice provided on strategies<br>- Increase enjoyment		Social support
**Burke, 2009, 2012; Ambeba, 2015**	Self-monitoring<br>Feedback<br>Goal setting			
**Carlson, 2012**	- Goal setting<br>- Self-monitoring<br>- Tailored feedback<br>- Goal revision			- Social support
**Carter, 2013**	- Goal setting (outcome)<br>- Self-monitoring<br>- Progress-monitoring<br>- Feedback			
**Castelnuovo, 2011, 2010**	- Goal setting<br>- Self-monitoring<br>- Feedback<br>- Relapse prevention	**-** Assign positive values to healthy behaviors and in aligning them with personal values;	- Stress management	- Cognitive restructuring

**Chambliss, 2011**	- Goal setting<br>- Self-monitoring,<br>- Feedback on progress<br>- Relapse prevention<br>- Coping planning	- Stress management	- Social support<br>- Stimulus control<br>- Cognitive restructuring<br>- Restructure environment
**Chung, 2014**	- Self-monitoring<br>- Feedback (on meeting recommendations)		
**Cussler, 2008**	- Progress-monitoring<br>- Feedback (weight progress)		- Social support<br>- Incentives
**Genugten, 2012**	- Goal setting<br>- Action planning<br>- Progress-monitoring<br>- Tailored FB<br>- Coping planning		- Social Support (peer –to-peer forum)
**Haapala, 2009**	- Goal setting<br>- Self-monitoring		
**Hersey, 2012**	- Goal setting<br>- Self-monitoring<br>- Tailored FB		- Social support (coaching)
**Johnson, 2008**			Social comparison<br>Feedback on reports
**Kirk, 2003, McConnon, 2007**	- Progress self-monitoring (personal goals)<br>- Tailored feedback		
**LaChousse, 2012**	- Feedback		

**Morgan, 2010, 2014; Collins, 2011**	- Goal setting<br>- Self-monitoring<br>- Feedback<br>- Implementation intention<br>- Goal Revision		- Increasing knowledge<br>- Barrier identification
**O'Brien, 2014**	- Goal setting<br>- Action Planning<br>- Self-monitoring and Feedback<br>- Progress feedback<br>- Goal prioritization<br>- Coping planning	- Plans according to participants' preferences	- Social support (forums, blogs, FB, email/phone contact)<br>Demonstration of behavior
**Patrick, 2009**	- Goal setting<br>- Action planning<br>- Self-monitoring and Feedback<br>- Progress monitoring<br>- Coping planning		
**Pellegrini, 2012**	- Goal setting<br>- Self-monitoring<br>- Feedback		
**Quintiliani, 2014**			- Providing information<br>- Social support (social media and SMS)
**Roesch, 2010**	- Goal setting<br>- Planning<br>- Self-monitoring;<br>- Relapse prevention<br>- Discrepancy behavior –goal<br>- Goal revision		- Providing information<br>- Stimulus control<br>- Positive self-talk<br>- Social support<br>- Self-reward<br>- Pros and cons<br>-Identify barriers
**Rothert, 2006**	- Goal setting<br>- Action planning		- Providing information<br>- Tailored messages (barriers)<br>- Social support (buddy system)

**Shapiro, 2012**	- Goal setting<br>- Self-monitoring<br>- Coping planning<br>- Feedback (progress and behavior)			- stimulus control
**Svetkey, 2008, 2014, Stevens, 2008, Brantley, 2008**	- Goal setting<br>- Action planning<br>- Self-monitoring<br>- Tailored feedback<br>- Coping planning/Relapse prevention	Motivational interviewing techniques		- Provide information<br>- Social support (bulletin board)<br>- Accountability<br>- Frequent use awards
**Tate, 2001**	- External goal setting<br>- Self-monitoring<br>- Feedback		- Stress management	- Provision of information<br>- Social support (bulletin board; interventionist)<br>- Stimulus control
**Tate, 2003**	- Self-monitoring<br>- Feedback (behavior)			- Reinforcement
**Thorndike, 2012**	- Goal setting<br>- Self-monitoring<br>- Progress evaluation (discrepancy between current behavior and goal),<br>- Feedback (behavior)			
**Ware, 2008**	- Goal setting<br>- Action planning<br>- Feedback<br>- Coping planning			- Social support<br>- Pros and cons
**Webber, 2010a,2010b**	- Goal setting<br>- Self-monitoring<br>- Goal revision	- Motivational techniques based on MI<br>- Reasons for Weight loss elicited		- Pros and cons<br>- Provision of information
**Webber, 2013**	- Self-monitoring diaries (behaviors)<br>- Problem solving			- Provision of information<br>- Restructuring the physical environment
**Werkman, 2010**	- Goal setting<br>- Self-monitoring/self-evaluation<br>- Feedback (progress and behavior)	- Provision of choice on modules accessed		- Provision of information<br>- Pros and cons

**Wier, 2009**	- Coping planning<br>- Lifestyle modification strategies (e.g., self-monitoring, goal setting),		- Support form counselor
**Womble, 2004**		**-** Diet designed to match needs, likes, and lifestyle	- Social support (on-line meetings that were moderated; bulletin board support groups; buddy system)<br>- Reminders about program<br>- Prescribed diet<br>- Tailored physical activity recommendations<br>- Cognitive restructuring
**Wylie-Rosett, 2001**	- Goal setting<br>- Self-monitoring	Provisions of choice (e.g. self-select goals, options on recommended topics)	
