# Supplementary material for: A Theory- and Evidence-Based Digital Intervention Tool for Weight Loss Maintenance (NoHoW Toolkit): Systematic Development and Refinement Study
Source: J Med Internet Res. 2021 Dec 3;23(12):e25305. doi: 10.2196/25305 (PMC8686406; doi:10.2196/25305)
Supplement: Multimedia Appendix 2 [file jmir_v23i12e25305_app2.pdf]

Summary of a feasibility study of NoHoW TK, submitted as part of a deliverable in the NoHoW Project (Evidence-Based ICT Tools for Weight Loss Maintenance)

NoHoW has received funding from the European Union's Horizon 2020 research and innovation programme under grant agreement No 643309

Methods

Participants and recruitment

Eligible participants were aged ≥18 years, able to travel to the University of Derby, able to follow written and verbal information in English, ability to access the internet, currently or have been overweight in the past, with at least one weight loss attempt, and interested in managing weight. They were recruited primarily by advertisement or invitation from University of Derby, Slimming World and the local surrounding area by word of mouth. Interested participants then contacted the research team via email to express their interest.

Objective

The feasibility study explored the user experience (e.g. acceptability) and technical maturity of NoHoW TK v1.0. The aim of the feasibility study was to identify and correct any technical- or user experience-related issues hindering the TK use and acceptance. Results from the study were used to revise the TK specification, as well as support integration of the TK into the overall ICT architecture and communications with the data hub before proceeding with the main randomised controlled trial.

Procedure and measures

The study participants used the TK for one month. Quantitative evaluation measures were collected using Qualtrics (an online survey system) and they included: a short demographic questionnaire, a short version of the Regulations for Weight Management questionnaire (adapted from the Regulation for Eating Behaviour Scale, REBS; Pelletier Dion, D'Angelo, and Reid 2004[1]), and user experience questionnaires, which were constructed based on the Technology Acceptance Model for Mobile Services. User experience questionnaires included '5-day user experience questionnaire', which measured first impressions after one week's use of the TK, and 'end of study user experience questionnaire', which measured the use experiences after the study period. In addition, a log of user enquiries and related issues with the Toolkit was kept, and Log files were collected automatically by the Toolkit. Qualitative data on user experiences were collected from a sub-set of participants through semi-structured interviews. Ethical approval was accepted by the Psychology Research Ethics Committee (PREC) of the University of Derby.

Data analysis

Quantitative data

1. Age and gender distribution were calculated from the short demographic questionnaire and also questions related to participants' previous weight loss attempts and needs were analysed.

[1] Pelletier, L. G., Dion, S. C., Slovinec-D'Angelo, M., & Reid, R. (2004). Why do you regulate what you eat? Relationships between forms of regulation, eating behaviors, sustained dietary behavior change, and psychological adjustment. Motivation and Emotion, 28(3), 245–277. doi:10.1023/B:MOEM.0000040154.40922.14

2. The scores were calculated from the Regulations for Weight Management questionnaire.
3. User experience questionnaires:
    a. The average scores were calculated from the user experience questionnaires.
    b. The average scores for the overall score given to the Toolkit, the likelihood to continue using the Toolkit and the likelihood of recommending the Toolkit were calculated for both the first impressions questionnaire and the end questionnaire.
    c. For each feature of the Toolkit, average ratings for importance, usefulness, ease of use, convenience, enjoyability, satisfaction, and motivation were calculated.
4. Log files: Various metrics describing usage activity were calculated based on the log files. For example, for each user, the number of logins, usage days and usage sessions were determined. The modules and sessions visited by each user were determined. In addition, session-specific ratings of usefulness, ease of use and enjoyability and voluntary comments provided by the users were extracted.

Qualitative data

Notes from the interviews were thematically analysed using NVivo 11 Pro software, which supports qualitative and mixed methods research. The purpose of the analysis was to explore the themes of the user experience of the Toolkit. The process was deductive content analysis process (Elo and Kyngäs, 2007) where an unconstrained matrix was used for the analysis. The main categories of the matrix were Benefits, Concerns and Improvement ideas related to the TK, but during the analysis three more categories were formed: Expectations, Motivation to use and Use context.

Results

In total, 37 eligible participants expressed interest in participating in the study and 24 participants completed the baseline questionnaire. The average age of the participants was 41 years (range 23-63) and the majority of the participants were women (n=22). Adherence to the study was low - 20 participants started using the TK, but only 14 of them completed the 5-day questionnaire and only 9 completed the 30-day questionnaire. The research group faced challenges with low study adherence, albeit the data collected was consistent and allowed for analysis needed for the feasibility study. Thus, data saturation was achieved with a smaller sample than estimated in the DoA - adequate information was obtained to develop TK V2.0. 4 participants were recruited for in depth interviews.

Participants had more positive attitudes toward the TK in the 5-day user experience questionnaire than in the end of study questionnaire. The benefits of the TK were related to usability, credibility and trust whereas more critical attitudes were presented toward the visual appearance of the TK. Participants also indicated that the TK did not fully meet their expectations. The overall score for the TK, using a scale from 0 to 10, was 5.44 (range 3-7). Intention to use it in the future was 4.22 (range 0-10) and recommendation of use to others rate 4.67 (range 2-7). Participants rated the different functionalities based on their importance, ease of use, convenience, enjoyability, satisfaction, motivation and usefulness. Regarding all functionalities, ease of use received the highest scores whereas motivation to use received the lowest. The feedback informed the consortium where to make improvements. As an example, TK's technical suitability to different browsers and devices was improved and animated videos with avatars were removed.

The interviewees indicated that the TK has potential, and that there is a need for it, but there are also certain improvements regarding both content and functionality that need to be considered during the development of the TK v2.0. The interviews showed that participants did not have many a priory

expectations about the TK. They did expect practical things that "make them think and do", as well as self-monitoring functionalities, including tracking weight and calories. They also expected to have support for the psychological side of weight management. The benefits identified by participants mainly related to the ease of use, content and experienced value. The TK was characterized as easy and intuitive to use. More specifically, participants stated that it is easy to revisit sessions and that back buttons and "ticks" after the user has completed a session are helpful. They also stated that the visual appearance of the TK was nice and simple.

Regarding the content, participants stated that the TK was trustworthy and professional. Participants noted that the content had a logical flow and progressing through it supports a learning process. At a more detailed level, participants liked the fact that there is a video at the beginning of each session and that the TK provides both transcripts to read and videos to watch. Participants especially valued short sessions and practical tasks.

Participants stated that web-based solutions could be helpful in weight loss management. One participant stated that the TK is very useful as he lost half a stone during the study. The main benefit identified was the new information that the TK provides for its users. There were several themes that the participants found useful, but mindfulness was the most interesting one for participants

Several concerns that could negatively influence on adherence to the intervention were identified. One of the main identified challenges was that many of the functionalities did not work with mobile devices with a touch screen. Challenges were also faced with the interactive exercises – they did not work smoothly or participants did not fully understand them. Regarding the content, participants stated that there is too much information on some pages, the language is too academic and it is not always easy to apply the information to everyday life. The avatars were also characterized as unpleasant. In addition, participants stated that the TK was not interactive enough and there were problems with certain functionalities, such as the check-in questionnaire and sliding scales. Regarding the images, audios and videos there were several problems related to their appearance and also their functionality.

The main findings of the study and the resulting design implications for the development of the next version of the TK are summarized in the following table.

**Finding**	**Design implication**
The content has a logical flow and going through it provides a learning process.	Although there will be more content, it is important to follow the same logical flow that has been proven to be useful for the users.
Participants liked the fact that there is a video in the beginning of each session. They also liked that the TK provides transcripts to read and videos to listen.	Transcripts and videos will form the main content of the TK also in the future, although there will be some improvements and more interactivity (see other design implications).
Participants valued especially short sessions and practical tasks.	Short sessions and practical tasks will be favored when possible. However, some contents require longer sessions.
**Interactive exercises didn't** always **work or participants** had difficulties **understand them.**	Implementation of the interactive exercises will be improved having special emphasis on their technical functionality, usability and understandability.
Participants wished for access the information they have filled in earlier.	Forms will show the previous answers whenever possible. Users can modify the fields and save

	new answers. This will improve usability, especially with touch screen devices.
The avatars were characterized as unpleasant.	Avatars will not be used.
Participants stated that there is too much information on some pages and the language is academic.	Long text chapters, such as the Generic content will be cut into smaller text sections that are easier to read and will be reorganized into a library. This information can be accessed anytime but will also be embedded in the sessions. Communication experts will be consulted to improve the language.
Regarding the images, audios and videos there were several problems related to their appearance and also their functionality.	Videos will be re-filmed using more energetic language and less clinical backgrounds. The video player will be upgraded for the main study, so that it will not suggest promoted videos.
There should be more possibilities to track information.	During the main trial, participants will use the TK with scales and activity monitors. Weight monitoring functionality will have a bigger role. Special sessions will be provided for users who are struggling with weight gain.
There should be more eating related information.	Eating related content will be favored when possible. However, best effectiveness will be achieved when also other content will be provided. This new content will have to respect the 2x2 factorial design of the study, thus some content, e.g. related to emotions will be developed in the emotion regulation arm, while motivation content will be available on the motivation and self-regulation arm.
The TK should be more interactive.	Users' motivation to perform exercises will be improved by providing the sessions through an interactive map feature, providing a journey metaphor throughout the intervention.
Overall appearance could be more professional.	Instead of using tabs, the user interface (UI) will be tile-based. They should work better with touch screen devices and they also give a more modern look and feel.
There are several minor details that influence on user experience.	Several minor issues will be addressed. As an example, the project name "NoHoW" will be explained, inconsistencies with text, video and audio content will be removed, and grammatical errors and typos will be corrected.
