# Supplementary material for: A Theory- and Evidence-Based Digital Intervention Tool for Weight Loss Maintenance (NoHoW Toolkit): Systematic Development and Refinement Study
Source: J Med Internet Res. 2021 Dec 3;23(12):e25305. doi: 10.2196/25305 (PMC8686406; doi:10.2196/25305)
Supplement: Multimedia Appendix 3 [file jmir_v23i12e25305_app3.pdf]

Summary of a qualitative analysis of support needs and usability of NoHoW TK, submmited as part of a deliverable in the NoHoW Project (Evidence-Based ICT Tools for Weight Loss Maintenance)
NoHoW has received funding from the European Union's Horizon 2020 research and innovation programme under grant agreement No 643309

Methods

Participants and recruitment

Eligible participants were adults aged ≥18, who had made a deliberate weight loss attempt in the past 2 years, had lost at least 5% of their body weight in the preceding 2 years, and had a pre-weight loss BMI of ≥25kg/m^2. There were no criteria regarding current BMI or regain/maintenance status, as the trajectory of WLM vs. relapse is such that many people who had previously lost weight, and therefore qualified for inclusion, would not have successfully maintained their loss. Several methods were used to ensure recruitment of a diverse sample. These included: asking local GP practices to approach potentially eligible patients; advertising on social media websites (e.g., Facebook and Twitter); and snowball sampling.

Interview topic guide

The Portuguese interview schedule was designed to focus, in more detail, on the motivational aspects of WLM, as well as explicitly eliciting support needs relevant to the use of online tools. In addition, Portuguese interviewees were shown a beta version of the NoHoW toolkit. This included: (1) a demonstration of the navigation procedures; i.e., the dashboard, self-monitoring features (tile system) and map, and a session that included a Qualtrics implementation, a video recording, and a whiteboard animation; and (2) a session of the Toolkit. In this session, users first saw a 'welcome' video recorded by a team member (duration: about 1 minute). Secondly, they completed a quiz (select and drop system) about 'myths and facts of weight loss maintenance' to test the embedded Qualtrics system. Lastly, they saw an animation video, "Eating: old brain, new rules", about the evolutionary approach to eating and obesity. Interviewees were asked to comment on aspects such as the content and modes of delivery, the layout and ease of navigation, the use of integrated technology, and issues around the timing and user experience of interacting with the programme.

Procedure

Interested participants in Portugal were asked to contact the respective research team in order to confirm their eligibility and book a time for the interview. Prior to the interview session, eligible

participants received the Participant Information Sheet by email and were given the opportunity to ask any questions.

Interviews were conducted at the university or, in case where the participant was unable to travel, a home visit or telephone interview was offered. At the commencement of the interview session, the details of the study were further explained and any questions were answered before participants provided their written informed consent. Interviews were conducted in the local language by two members of the NoHoW research team, with relevant qualifications (e.g., psychology) and experience in qualitative data collection and analysis. The number of interviews conducted was determined by cumulatively reviewing interviews and determining when data saturation for each of the key research questions had been reached.

Interviews lasted approximately an hour, and were audio recorded and then transcribed verbatim. Transcription, coding, and analysis were all conducted in the local language, with translation into English being completed for the purposes of writing the final report.

Approval from the relevant research ethics committees at the University of Lisbon was obtained prior to recruitment and data collection. At the conclusion of the interview, participants were fully debriefed and given the opportunity to ask any further questions about the study or how their data would be used.

Coding framework for qualitative data

The Portuguese analysis was conducted in MAXQDA (version 12). Initially, using a top-down approach, transcripts were coded for a series of pre-defined themes, which differed according to the content of the interviews across the three countries. In each case, coding frameworks were defined a priori by the research teams based on the literature and previous theory and evidence that informed the development of the respective interview topic guides. Themes and sub-themes reflected constructs drawn from motivation theory, around which the motivational content of, and engagement with, the toolkit is based: competence (skills/mastery, and challenges and barriers); autonomy (including enjoyment, and perceived choice/ownership); motives/goals (intrinsic vs. extrinsic); and relatedness (including perceptions of support vs. pressure from health care professionals, and sources of social support).

Secondly, a bottom-up, thematic analysis was conducted to identify additional themes and links within each of the pre-defined thematic categories. The analysis of the section of the interviews that focused on user-experiences and feedback on the beta version of the toolkit was not explicitly guided by theory or previous evidence, and so was coded solely using a grounded, data-driven approach. This part of the analysis followed standard qualitative procedures and included becoming familiar with the data, generation of initial codes, identifying themes among codes, reviewing the themes, and defining and naming the themes. Following familiarisation with the data and discussions about emerging themes and potential codes, coding was completed independently by two researchers. Final agreement on coding and resolution of any disagreements were achieved through discussion between the research team, including at least one team member who had not been involved in the conduct or initial coding of interviews.

Results

Descriptive characteristics for the Portuguese participants

ID	Age	Gender	Pre-weight loss BMI (kg/m^2)	Weight loss (%)	Current BMI (kg/m^2)
1	39	M	44.4	18.8	36.1
2	46	M	43.2	43.4	24.4
3	50	F	30.9	18.3	25.2
4	57	F	32.0	25	23.3
5	25	F	26.1	23.6	20.0
6	32	F	31.2	32.7	27.6
7	40	F	35.2	14.4	30.1
8	23	F	27.3	25.3	20.4
9	23	M	31.4	32.7	21.1
10	38	F	28.7	14.1	24.6
11	38	F	27.0	10.0	24.3
12	38	F	28.7	14.1	24.6

Thematic analysis

Mode of access to the TOOLKIT, self-monitoring tools and interface with NoHoW toolkit

All participants, with the exception of PT participant 6, reported no obstacles to the use of the NoHoW toolkit. The design mockup was clearly understood by all and participants felt it was intuitive, simple and engaging: "The tiles are more engaging, more direct. People with mobile phones use everything with tiles nowadays, they are much more familiar" (PT participant 1); "It is pretty much straightforward. Easy to understand and navigate, and it looks cool" (PT participant 9).

The ease of access from any type of mobile devices or computer was seen as an advantage by all participants, although there was no clear preference for a particular device to access the toolkit. Further, access to the Internet wasn't considered to an obstacle to the use of the toolkit as everyone could easily access it. Likewise, participants did not mention any obstacles to the use of the fitness tracker (Fitbit) during the intended study period (18 months). Even when asked about the requirement for weekly battery charging and data synchronisation, participants reinforced their willingness to use it, indicating that it would be an easy procedure to integrate into their daily routines. Some participants anticipated that they might have some difficulties in maintaining a daily weighing routine, but all said that the commitment of weighing themselves several times per week would be reasonable.

Again, with the exception of Participant 6, who stated: "I don't have any desire to participate. I'm

being honest. None. It's too long. And it would become an obligation… I think that in my case, this digital system wouldn't do much difference. For me I would prefer a thousand times to spend my money on a nutritionist, to have a real person, a support…. Instead of a virtual support system", all other participants considered the study period to be feasible and said they would be willing to commit for that period if they were participating.

The preferred schedule for accessing the toolkit was in the evenings, in the comfort and quietness of home, but overall participants considered it not to be a problem receiving prompts at other times of the day. Even when they were asked about constraints in accessing the toolkit outdoors, participants stated that they could easily manage using the internet on their mobiles and could use headphones for videos and content with audio. Regarding the weekly time commitment, all participants said that they had no restraints or problems, considering 5-10 minutes every week to be perfectly manageable.

Self-Monitoring Tools

Regarding the use of self-monitoring devices, almost all participants could see their utility and were willing to use them. The duration of the program (18 months) was not perceived as a barrier for their use. PT participant 6 was the only one who stated that it would not be feasible to use the fitness tracker for the 6 months of the active intervention, or to commit to daily weighing, because she was already in the routine of weighing herself only every two weeks, and used her own scales: "Well, I think that I would continue to weigh using my scales, because I like them… I'm already regulating myself using that one, I want to continue, right?" Some participants mentioned that it would be hard to use the scales during periods of short vacations or other travel. Travelling abroad could also present problems with internet access, which is required to access the toolkit and synchronize data from the fitness tracker.

Toolkit Content Characteristics – Content engagement, utility and extra support

From the interviews, it was concluded that all participants felt that the toolkit content was very acceptable. Regarding the type of videos that the toolkit contains, the use of whiteboards, animations, and the videos with a team member were unanimously approved. Also, the duration of videos (approximately 1-2 minutes) was considered reasonable by all participants.

The whiteboard/animation feature was considered the most appealing and engaging in terms of both the graphic design and content: "...I think I prefer the drawing hand. In my opinion, the hand is more engaging, it has a lot of movement" (PT participant 2). On the other hand, the video recorded with a team member was also perceived as having the advantage of being more relational and closer to the user: "the video...yes… it is a more personal approach, it feels that there is a relation" (PT participant 9); "And the video has another advantage, it gives the feeling that it is a more personal thing, more intimate" (PT participant 8); "Both complement each other, I think both… so I think both are good" (PT participant 11).

When asked about the necessity of having written support to accompany the video content, participants felt that this feature would not be crucial. PT participant 8 proposed a mixed system: "But I think that if it could be possible, for example if the application allowed you to choose between the options - video, audio and written content, it would be an advantage. Because one could for example want to see the video in places where is not suitable to see that…".

The interactive toolkit activities that were shown during the beta demonstration were perceived to be simple and useful by all participants. They did not anticipate problems in adjusting to the type of

tasks requested by the toolkit (e.g., quizzes, reflections): “It’s not complicated, I think it’s easy” (PT participant 2).

The possibility of including brief testimonies of people who had been successful in WLM was also something that all participants were very pleased with, even if it was presented in the format of some text accompanying an image: ““Yes it seems good to me, I think that the good examples are always good to hear, everybody likes those I think” (PT participant 1); “I think that if a person feels that reading a testimonial is motivating, I think it is worth including. Even for users that think that testimonial will not influence their motivation, just by reading or looking at the pictures, it will be retained in their mind and make a difference” (PT participant 8).

The Extra Support system is an alert system that is activated when the users reach the set limit of 3% above their initial weight, providing an extra session in the toolkit with targeted and relevant content and supporting tasks to better cope with these situations. All participants considered this aspect of the toolkit to be important and useful: “I think it would be useful, it is one more way to warn that it is time to change behaviours” (PT participant 2). The fact that this system could generate additional prompts wasn’t perceived as a potential obstacle to its use (i.e., it was not perceived as being intrusive).

In general, all participants felt that the toolkit was well designed, and although it was still in the development phase, they also predicted a good adaptation to the system: “In my case, I don’t foresee any difficulties, it is engaging, presents adequate information considering its aim” (PT participant 2); “I think it is useful, yes I think that the application is exceedingly well designed and exceedingly well done so far” (PT participant 8). Participant 5 described the utility of such a tool in supporting weight management: “Yes, I think that this is good for those people that already want to change their life or that already lost or just begun [to lose weight]” (PT participant 5).

Impact: User engagement with the toolkit

Key finding: It was concluded from the interviews that *the toolkit interface, navigation system (e.g., scroll-down, tiles) and different components (e.g., self-monitoring tools, support system) were well accepted by the target population* of the NoHoW intervention, and were considered engaging and easy to use. This last aspect seems to be very important for participants who stated that other digital tools sometimes involved complicated features. Media content, especially the animation videos (whiteboard), were well accepted by all participants interviewed; all agreed that these were highly engaging.

Implications: These findings offer support for the use of approximately 12 whiteboard animations in the NoHoW toolkit modules. The toolkit will present testimonials (rated as highly useful) from people of different ages and genders, and with various weight management experiences. Testimonials are considered useful to foster competence and relatedness. Finally, self-monitoring tools are a key component of the toolkit, providing information about the weight scales and activity trackers (steps and sleep), with several visualisation options (e.g., graphs per week, month).

Key finding: The Extra Support system (activated when users are struggling to keep their weight off/are showing evidence of weight regain) in the arms that include the Motivation and Self-Regulation components is another component of the toolkit that users considered to be very useful and engaging.

Implications: These results further substantiate the importance of providing such content for use in the event of weight regain. The Extra Support system will be activated when a specified weight upper limit is reached. When active, the user will be prompted to visit specific sessions (e.g., their favorite sessions, sessions with activities to cope with weight regain situations), will be asked to reflect on the strategies that have worked before to manage her/his weight, will have access to practical and tailored strategies (e.g., coping plans), and will be prompted to self-monitor more frequently. The Extra Support can be interrupted by the participant at any time, or when her/his weight decreases below the set upper limit.

Key finding: The *main obstacles* to the use of the toolkit envisaged by participants were: the use of the self-monitoring equipment (e.g., activity tracker or weighing scales) and internet access while traveling.

Implications: The user-toolkit communications component is set to facilitate the use of the activity trackers and weighing scales. Email prompts will be sent when irregular usage of the devices is noticed, providing the participant with options to improve engagement. At the beginning of the intervention (2nd visit to the research centre), participants will have a face-to-face training session on the use of the toolkit and devices. In this training session users will learn how to use the toolkit and devices (activity tracker, weighing scale), and how to deal with the most foreseeable difficult scenarios. Among these, the sustained use of the devices, travelling, and internet issues will be dealt with.

Extraction of Interview Topic Guide

Construct	Questions/Interview Themes	Source literature/ gap in knowledge	Desired outcome	Notes
Toolkit Characteristics	Interviewer introduces the TK beta version and asks the participant to explore it for a while (e.g. 5 mins). Afterwards, the interviewer says: "I would like to hear what you think of these elements – what would be helpful/attractive to you and what would not (ask for each element):<br>• user engagement;<br>• Graphism;<br>• Easy of navigation trough menus of toolkit;<br>• Easy of access to key features and information (graphs; etc)<br>• Preferable platform for program delivery (mobile devices vs home computer)<br>• Tasks are clear and easily understandable<br>• Internet access (depending on content)<br>• Types of preferred videos/implementations: Real characters with whom they can identify (age, gender; real stories WL); Team members; Animations (team members avatar or identified characters)		To understand suitability of Toolkit technical features.	Toolkit beta version and screenshots will be used
Program Characteristics	Imagine you are participating in a WLM program like this one. – Would like to have a fixed schedule to assess the toolkit or chose the day/time, and be able to change when needed.<br>- Would you prefer to use it/be engaged in critical moments (lunch; snack time) throughout the day or in more relaxed "confort moments"		Personal preferences concerning program delivery	Toolkit beta version and screenshots will be used

	(at home, end of the day; weekend - Few but longer Sessions; Weekly)<br>- Do you like the idea of additional prompts when relevant changes occur, for example on your weight? User tailored (frequency, time, content) or standardized prompts?<br>- What do you think about time prerequisites - Session duration and frequency - What would be for you the optimal session duration? And number of sessions per week?<br>- How do you think you would manage this program in Holidays/travelling? (travelling abroad/Christmas)<br>- What do you think about the total duration of the program (about 6 months). If not satisfied, what you consider adequate for the total duration of program like this one?			
