# Supplementary material for: A Theory- and Evidence-Based Digital Intervention Tool for Weight Loss Maintenance (NoHoW Toolkit): Systematic Development and Refinement Study
Source: J Med Internet Res. 2021 Dec 3;23(12):e25305. doi: 10.2196/25305 (PMC8686406; doi:10.2196/25305)
Supplement: Multimedia Appendix 4 [file jmir_v23i12e25305_app4.pdf]

Multimedia Appendix 4

Table S1. Theoretical guiding principles underlying intervention design – Motivation and Behaviour Regulation Arm

Address Basic Psychological Needs/Increase Autonomous motivation
**Promote autonomy**
1. Offer choice on how users engage with the toolkit and implement the content provided –specific behavioural (physical activity and/or dietary) and weight goals are formulated and planned by the individual; type of strategies used; contact (e.g. frequency of contact, day of the week, additional prompts, data users feed to the toolkit – personal notes); content delivery;<br>2. Use non-directive language (avoid “shoulds”, “musts”, “we recommend”);<br>3. Elicit user’s views on condition/ behavior, and personal exploration of behaviors;<br>4. Provide rationale and/or evidence for behavioral changes and then let users decide if they want to follow it (diminishes resistance).<br>5. Prompt reflection on current strategies regarding origin, target/goal, and sustainability. Prompt reflection exercises on the (physical and psychological) changes associated with changes in weight and related- behaviors;
**Promote relatedness**
6. Provide feedback - reciprocity (whenever possible immediate feedback);<br>7. Use judgment- and bias-free language denoting regard and respect for person at any weight; adopt empathetic tone and use obesity-relatable examples.<br>8. If appropriate, ask permission or allow to opt-out before providing direct advice;<br>9. Give users the option to contact the research team for additional support;<br>10. Use testimonials stories related with WLM (e.g. personal reasons; successful overcoming barriers, modeling, etc.) close to the participant experience.<br>11. Personalize messages (e.g. use first name, tailored to user experience whenever possible).
**Promote competence**
12. Provide an easy structure from where to choose from (e.g. use multiple choices/drop-down boxes; select goals from a list of common strategies for WLM).<br>13. Use testimonials stories related with WLM (e.g. personal reasons; successful overcoming barriers, modeling, etc.).<br>14. Provide information (e.g. misconceptions) of WLM and related behaviors, using credible sources;<br>15. Encourage maintaining strategies already used by participants if these are sustainable.<br>16. Assure that physical access to the intervention content fits with users’ daily routines and, where appropriate, typical usage of digital devices.<br>17. Promote selection of optimal challenging goals and promote the use of SR skills (see principles to promote SR capacity below)
**Promote self-regulation capacity and skills**
18. Encourage and provide support on the formulation and planning for specific behavioral (physical activity and/or dietary) and weight goals, against which users can assess their performance.<br>19. Encourage regular self-monitoring of behavioral and weight goals, by providing wirelessly connected scales, accelerometer, dietary intake diary, and goal progress assessment.<br>20. Provide weekly feedback on behaviors and weight goal progress (promotes reflection on personal progress and use of additional self-regulation strategies when needed).<br>21. Provide users with the opportunity to revise and plan new goals, whenever goals are achieved or found to be too difficult.<br>22. Encourage and provide support on the formulation of coping plans (identification of difficult situation and strategies to avoid/deal with it).<br>23. Additional prompts/contact with users can facilitate individual self-regulation, by emphasizing revisiting specific modules, goal revision, and problem solving.

Table S2. Intervention techniques included in Motivation and Behaviour Regulation Arm

BCTs	MBCTs
Discrepancy between current behaviour and goal (BCT v1_1.6)<br>Problem Solving (includes 'Relapse Prevention' and 'Coping Planning'; BCT 1.2)<br>Goal setting outcome (BCTv1_1.3)<br>Action planning (BCTv1_1.4)<br>Credible Source (BCTv1_ 9.1)<br>Prompt focus on past success (BCTv1_15.3)<br>Self-monitoring of behaviour (BCTv1_2.3)<br>Self-monitoring of outcome (BCTv1_2.4)<br>Reduce negative emotions (BCT 11.2)<br>Social support (emotional; BCT 3.3.)	Explore perspectives on condition or behaviour (MBCT1)<br>Explore potential sources of pressure for behavior change (MBCT2)<br>Explore life aspirations and values (MBCT4)<br>Provide a meaningful rationale (MBCT5)<br>Provide choice (MBCT6)<br>Facilitate autonomous goals or outcomes (MBCT7)<br>Explore intrinsic rewards (MBCT8)<br>Encourage the person to be supportive towards others with a similar condition (MBCT9)<br>Acknowledge and respect perspectives (MBCT10)<br>Acknowledge feelings (MBCT11)<br>Address obstacles for change (MBCT18)<br>Clarify expectations (MBCT19)<br>Assist in setting optimal challenge (MBCT20)<br>Offer constructive, clear and relevant feedback (MBCT21)<br>Help develop a clear and concrete plan of action (MBCT22)<br>Promote self-monitoring (MBCT23)<br>Explore sources of support from others (MBCT24)<br>Explore ways of dealing with pressures (MBCT25)

Note. BCTTv1: Behaviour Change Techniques Taxonomy version 1 [1]; MBCT: Motivation and Behaviour Change Techniques [2].

References

1. Michie S, Richardson M, Johnston M, Abraham C, Francis J, Hardeman W, Eccles MP, Cane J, Wood CE (2013) The behavior change technique taxonomy (v1) of 93 hierarchically clustered techniques: building an international consensus for the reporting of behavior change interventions. Ann Behav Med 46:81–95

2. Teixeira PJ, Marques MM, Silva MN, et al (2020) A classification of motivation and behavior change techniques used in self-determination theory-based interventions in health contexts. Motivation Science. https://doi.org/10.1037/mot0000172
