# Supplementary material for: A Theory- and Evidence-Based Digital Intervention Tool for Weight Loss Maintenance (NoHoW Toolkit): Systematic Development and Refinement Study
Source: J Med Internet Res. 2021 Dec 3;23(12):e25305. doi: 10.2196/25305 (PMC8686406; doi:10.2196/25305)
Supplement: Multimedia Appendix 5 [file jmir_v23i12e25305_app5.pdf]

Multimedia Appendix 5

Table S1. Theoretical guiding principles underlying intervention design – Emotion regulation

Address emotion regulation
**Psychoeducation: Contextual and internal cues of over-eating and emotional eating**
1. Rationale of the evolutionary basis of eating behavior and weight management.<br>2. Rationale on emotion regulation systems according to evolutionary analysis and affective neuroscience research and theory.<br>3. The interactive relationship between evolved emotion and motivational systems and their impact in current adaptive vs. maladaptive behaviours.<br>4. The multiple and interactional functions of eating behaviour (physiological, social, emotional).<br>5. Emotional eating as the tendency to overeat in response to negative emotions.<br>6. Affect-regulation models of overeating.<br>7. Shame-pride model of eating psychopathology.<br>8. Biopsychosocial evolutionary model of eating psychopathology.<br>9. The role of difficulties in distinguishing hunger sensations from physiological cues linked to emotional states - Psychosomatic theory.<br>10. Eating as a way to search for comfort and tone down distress by increasing positive emotions.
**Mindfulness: Promote contact with present moment and eating awareness**
11. Mindfulness as a cognitive process that involves bringing awareness to the present moment, nonjudging and accepting the experience.<br>12. Decentering and self-monitoring to promote meta-cognitive awareness of internal present-moment experience.<br>13. Mindfulness as a process of attention control, involving a curious, nonjudging approach to the ongoing external and internal experience (thoughts, emotions, sensations).<br>14. Mindfulness as a trait that can be cultivated and developed to promote a less reactive relationship with the present moment experience.<br>15. Mindfulness training to promote distress tolerance, acceptance of aversive experiences, wellbeing, and diminish emotional distress.<br>16. Eating as an object of mindful attention.<br>17. Mindful intuitive eating to promote: an increased awareness of internal cues of hunger, fullness and satiety; an increased awareness of and diminished reactivity to external and emotional precipitants of (over)eating – conceptual principles of Intuitive eating.
**Compassion: Promote self-compassion and compassion to others and diminish shame and self-criticism**
18. Biopsychosocial evolutionary model of shame in the context of competition for social attractiveness; weight stigma.<br>19. Conceptualization of the two dimensions of shame: external and internal shame.<br>20. Conceptualization of compassion within Compassion Focused Therapy as involving sensitivity to and mindful awareness of emotional distress, with the understanding of its causes (evolutionary, contextual) and the motivation to alleviate it.<br>21. Soothing rhythm breathing and compassionate imagery exercises to promote self-compassion and compassionate actions.
**Values-based action: Promote action towards personal values**

22. Acceptance and Commitment Therapy model of psychological (in)flexibility. 23. Values as embodied intentions for how one wishes to act in personally relevant domains (e.g., family, friendship or romantic relationships, professional, social, spiritual). 24. Committed action: promote the engagement in meaningful actions to live a life of meaning, purpose and vitality with actions that are congruent with freely chosen personal values. 25. Compassionate intention to engage in helpful actions towards wellbeing.

Table S2. Intervention techniques included in Emotion Regulation Arm

Intervention techniques
Psychoeducation on compassion [1-5]
Psychoeducation on emotional eating [5]
Creative hopelessness [6, 7, 10]
Mindful eating [8]
Definition of goals and values-based actions. [6, 7, 11]
Psychoeducation on mindlessness and mindfulness [8, 9, 12]
Commitment to values-based living [6, 7, 11]
Psychoeducation on evolutionary-based motives underlying difficulties to regulate eating behaviour and weight.
Psychoeducation on sociocultural pressures to achieve a specific physical appearance within an obesogenic environment.
Psychoeducation on the role of eating behaviour as a form of emotion regulation
Psychoeducation on the role of shame and self-criticism
Decentering
Mindfulness of the breath
Bodyscan meditation
Mindfulness of thoughts
Mindfulness of emotions
Mindful eating
Decentering
Soothing rhythm breathing
Psychoeducation on lapses and relapses
Compassion for the self-meditation
Compassionate other meditation
Compassionate self for self-criticism meditation

References:

1. Gilbert, P. (2005). Compassion: Conceptualisations, research and use in psychotherapy. New York: Routledge.

2. Gilbert, P. (2009). Introducing compassion-focused therapy. Advances in psychiatric treatment, 15(3), 199-208.

3. Gilbert, P., & Choden. (2013). Mindful compassion. London, UK: Constable-Robinson.

4. Gilbert, P., & Irons, C. (2005). Focused therapies and compassionate mind training for shame and self-attacking. In P. Gilbert (Ed.), Compassion: Conceptualisations, research and use in psychotherapy (pp. 263–325). London: Routledge.

5. Goss, K., & Gilbert, P. (2002). Eating disorders, shame and pride: A cognitive–behavioural functional analysis. In P. Gilbert & J. Miles (Eds.), Body shame: Conceptualisation, research and treatment (pp. 219–255). New York: Brunner Routledge.

6. Hayes, S. (2004). Acceptance and commitment therapy, relational frame theory, and the third wave of behavioral and cognitive therapies. Behavior Therapy, 35(4), 639-665. doi:10.1016/s0005-7894(04)80013-3

7. Hayes, S., Strosahl, K. D., & Wilson, K. (1999). Acceptance and Commitment Therapy: An experiential approach to behavior change. New York: Guilford Press.

8. Kristeller, J., & Wolever, R. (2010). Mindfulness-based eating awareness trainig for treating binge eating disorder: The conceptual foundation. Eating Disorders: The Journal of Treatment & Prevention, 19(1), 49-61. doi:10.1080/10640266.2011.533605

9. Segal, Z., Williams, J. & Teasdale, J. (2002). Mindfulness based cognitive therapy for depression: A new approach to preventing relapse. New York: Guilford Press.

10. Tangney, J., & Dearing, R. (2002). Shame and Guilt. New York: Guilford.

11. Tirch, D., Schoendorff, B., & Silberstein, L. R. (2014). The ACT practitioner's guide to the science of compassion: Tools for fostering psychological flexibility. New Harbinger Publications.

12. Tribole, E., & Resch, E. (1995). Intuitive eating: A revolutionary program that works. New York: St. Martin's Griffin.
