# Supplementary material for: A Theory- and Evidence-Based Digital Intervention Tool for Weight Loss Maintenance (NoHoW Toolkit): Systematic Development and Refinement Study
Source: J Med Internet Res. 2021 Dec 3;23(12):e25305. doi: 10.2196/25305 (PMC8686406; doi:10.2196/25305)

Multimedia Appendix 6. Graphical view of a Tile: Weight Tile

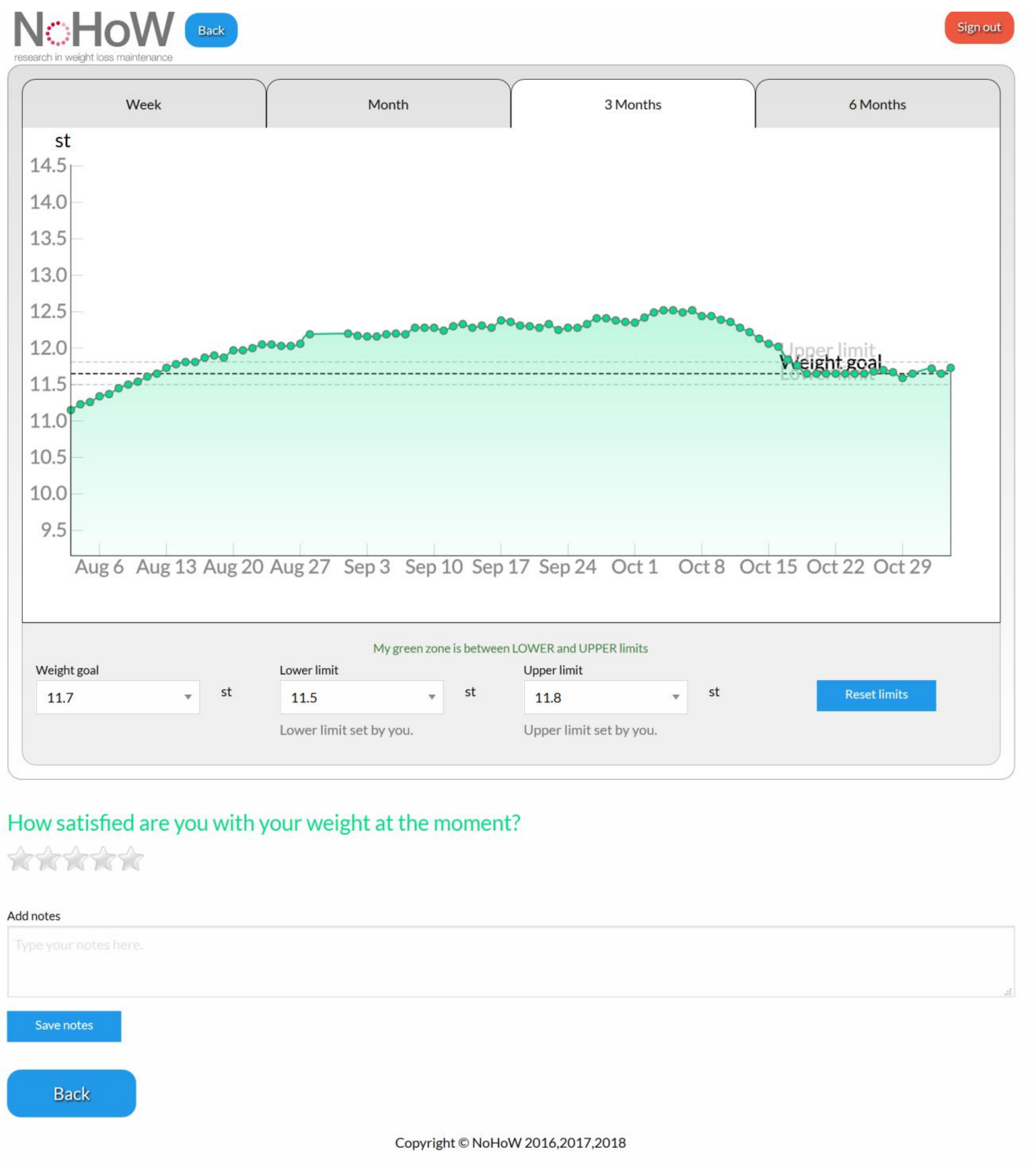

Supplement: Multimedia Appendix 6 [file jmir_v23i12e25305_app6.pdf]
