# Supplementary figures and images for: A Theory- and Evidence-Based Digital Intervention Tool for Weight Loss Maintenance (NoHoW Toolkit): Systematic Development and Refinement Study
Source: J Med Internet Res. 2021 Dec 3;23(12):e25305. doi: 10.2196/25305 (PMC8686406; doi:10.2196/25305)

## Multimedia Appendix 7. Personal Route-Map

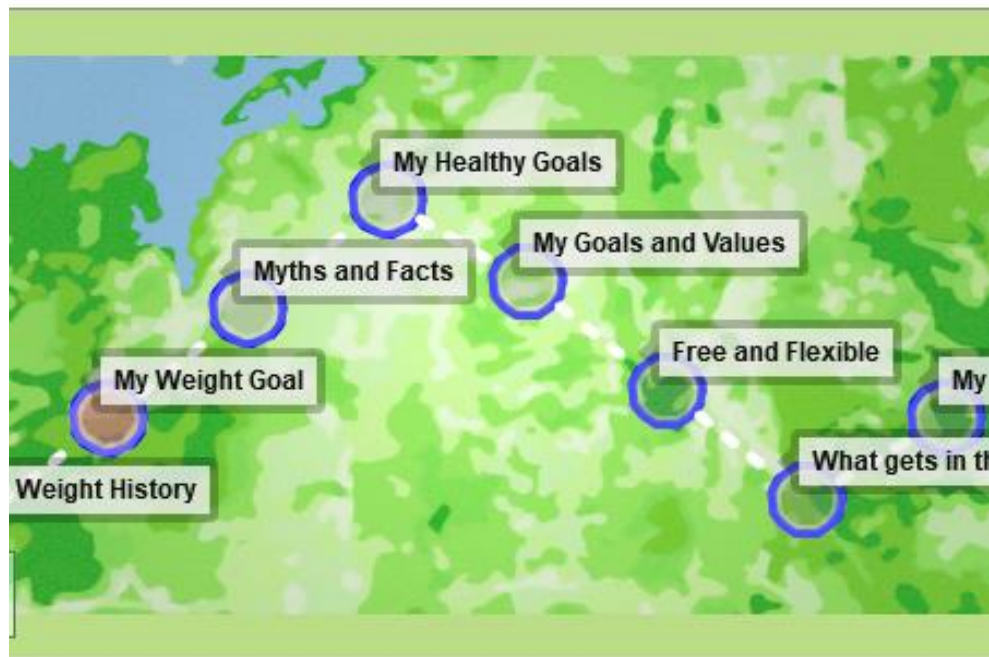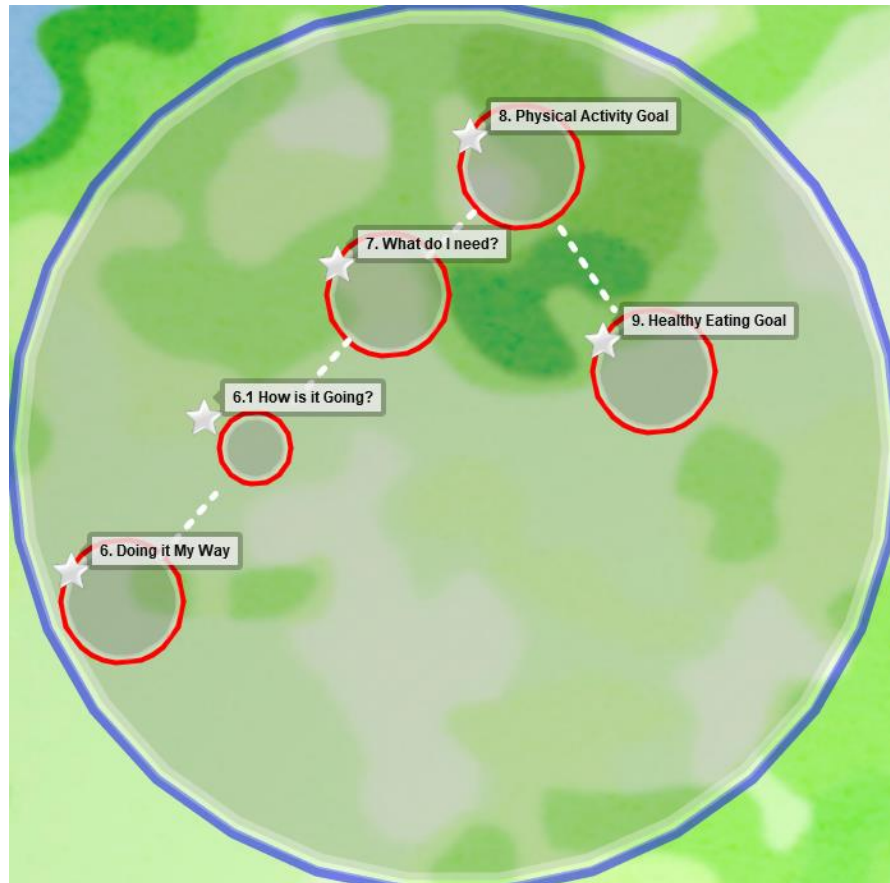

Supplement: Multimedia Appendix 7 [file jmir_v23i12e25305_app7.pdf]

► Shoulds vs Wants

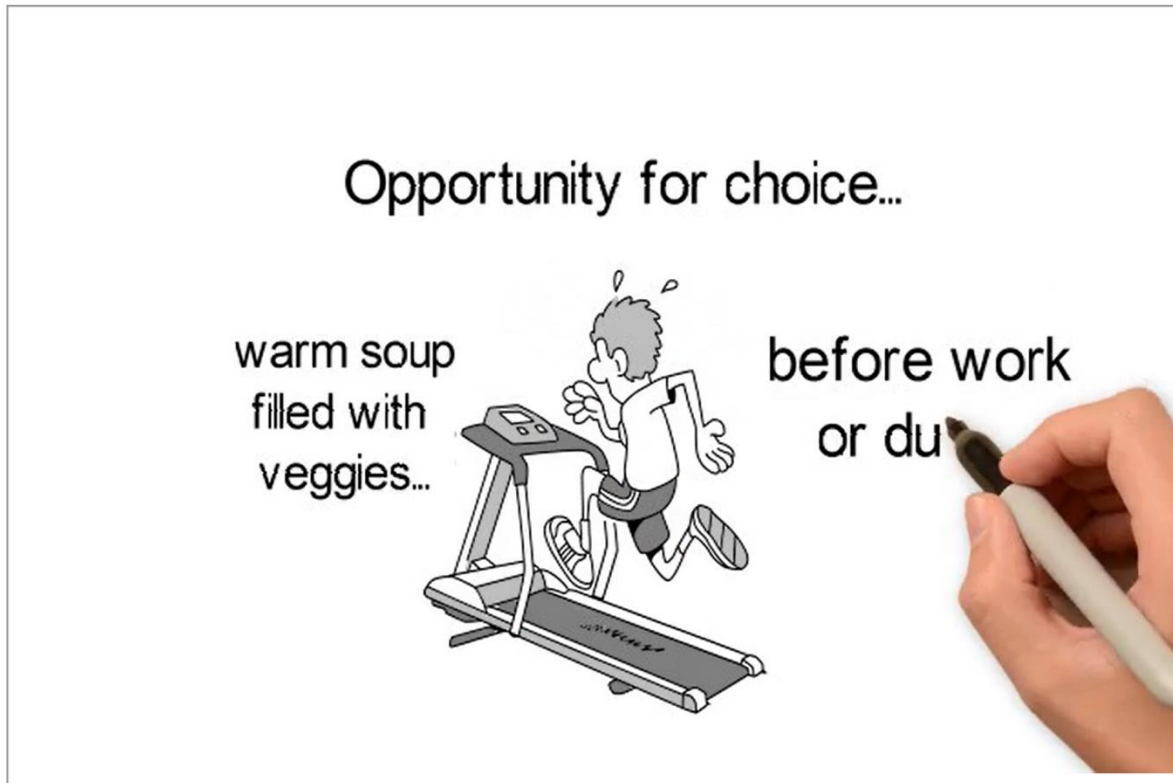

## ► Shoulds vs Wants

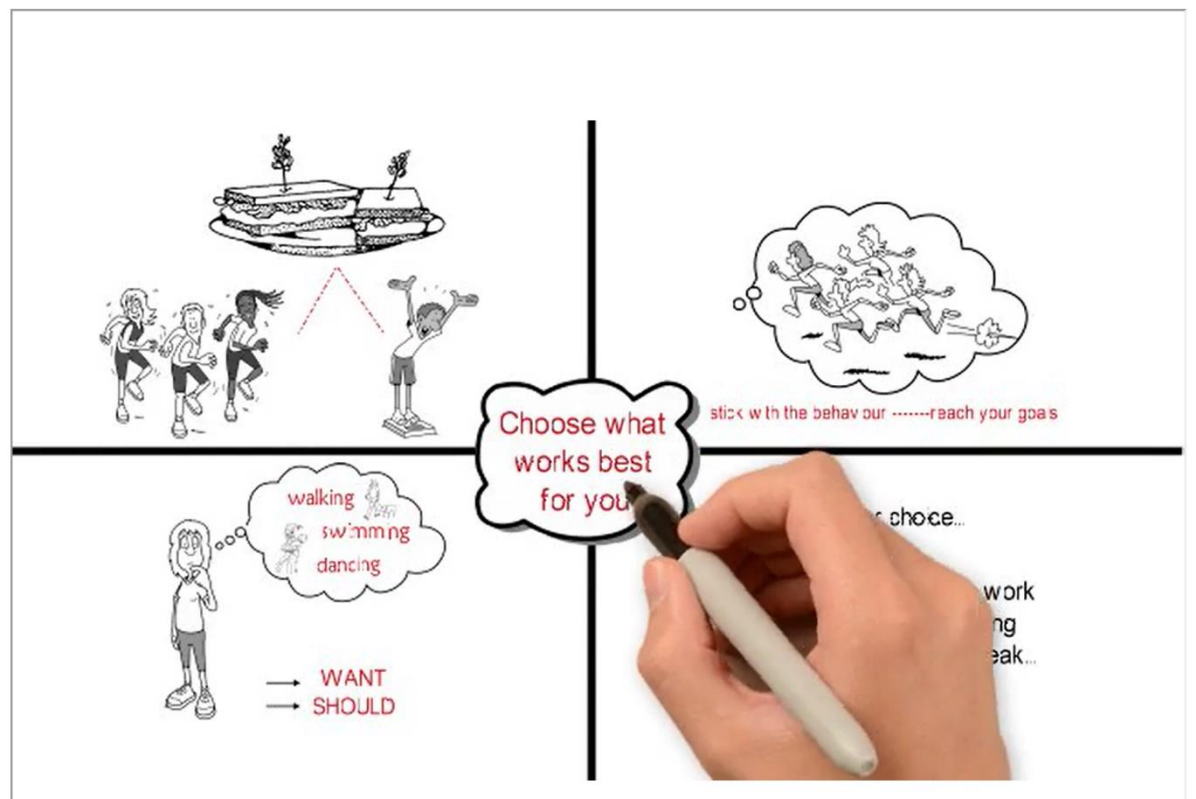

Supplement: Multimedia Appendix 9 [file jmir_v23i12e25305_app9.pdf]
