# Supplementary material for: A Theory- and Evidence-Based Digital Intervention Tool for Weight Loss Maintenance (NoHoW Toolkit): Systematic Development and Refinement Study
Source: J Med Internet Res. 2021 Dec 3;23(12):e25305. doi: 10.2196/25305 (PMC8686406; doi:10.2196/25305)
Supplement: Multimedia Appendix 8 [file jmir_v23i12e25305_app8.pdf]

NoHoW
research in weight loss maintenance

NoHoW has received funding from the European Union's Horizon 2020 research and innovation programme under grant agreement No 643309

Table 1. Motivation + Behaviour Regulation Toolkit – Modules Structure

Module 1 - My weight history
2 sessions

Sub-modules	Key points	Target Theoretical Constructs	Intervention techniques	Rationale	General Description of Implementations
1.a. "Looking back"	1) Review weight change history: weight trajectory, characteristics of previous weight changes (strategies and feelings associated).	- Self-awareness<br>- Competence	-Explore perspectives on condition or behavior (MBCT1)<br>-Monitoring of emotional consequences (close to BCTTv1 5.4.)	- By reflecting on past experiences of weight changes (specially on triggers, and emotional aspects), it increases self-awareness on the relation between weight changes and the core assets of the self. Self-awareness is a dimension of autonomy.	First, users are presented with a set of questions about important weight changes that occurred throughout their adult life – (1) highest weight, (2) lowest weight, (3) weight at which felt best, (4) weight at which felt worst. This information will appear in a "weight life line".
			-Prompt focus on past success (BCTv1 15.3)	- By identifying previous successes and strategies that were associated with important weight changes (loss and regain), it increases knowledge and confidence in what one could engage in to better manage personal weight (Competence).	Next, users select from a list the strategies/approaches they consider to be more associated with successful WL and with weight gain.
			- Provide choice (MBCT6)	-Provide guided choice (autonomy) based on successful models – strategies that worked with successful weight maintainers	Finally, users are prompted to check a wordle with commonly reported strategies for weight management success. (short session)
1.b."My strategies"	2) Identify weight loss strategies/ approaches used in the last WL attempt and assess their sustainability in time	- Self-awareness<br>- Competence<br>- Self-efficacy	- Prompt focus on past success (BCTv1 15.3)<br>- Provide a meaningful rationale (MBCT5)	-By reflecting on the utility and sustainability of each strategy/practice for WL/WLM and on its distinct characteristics (-integrated in daily life, and linked to deeper values), participants can be more aware of what fits them best.	First, users are asked to identify the sustainability of the strategies for the last WL attempt – 1a. (*green or yellow* according to the potential for *sustainability)*.
			- Explore life aspirations and values (MBCT4)	- Promotes perception of efficacy by providing successful examples. Highlights the link between successful strategies with and internal coherence deeper values and meaningful goals.	Next, a testimonial presents an example of a sustainable practice in two perspectives: 1) integrated in daily routine and 2) congruent with personal deeper values.
			- Explore intrinsic rewards (MBCT8)	-Encourage to try out new activity and explore what could be fun and enjoyable (autonomous action via intrinsic motivation).	Finally, users are prompted to choose one strategy they are willing to try (short session).

Module 2 – My weight goals
2 sessions

Sub-modules	Goals	Theoretical constructs	Intervention techniques	Rationale	General Description of Implementations
2.a "How am I doing?"	1) Learn about self-monitoring and self-referenced feedback, its role on with goal setting/revision, and reflect on individual options and preferences	- Self-regulation: monitoring, seeking feedback, awareness of standards and actual behaviour<br>- Competence, self-efficacy<br>- Autonomy (ownership)	- Self-monitoring of behavior (BCTv1_2.3) and of outcome (BCTv1_2.4)<br>-Promote self-monitoring (MBCT23);<br>- Offer constructive, clear and relevant feedback (MBCT21);<br>-Discrepancy between current behavior and goal (BCT v1_1.6);<br>-Provide choice (MBCT6);<br>-Help develop a clear and concrete plan of action (MBCT22)	- By prompting individuals in taking interest in current health behavior as source of information for goals, and seeking self-referenced feedback, can help individuals to be more aware of their progress towards their goals (mean to increase awareness of standards and behavior).	Participants first read a testimonial about the importance of monitoring PA and Eating Behavior for weight maintenance.<br><br>Next, a whiteboard animation on self-monitoring tools is presented introducing the health graphs available in the NoHoW TK Dashboard. It is highlighted the importance of using self-monitoring tools to settle goals, keep track of progress and adjust them.
				- Awareness of multiple choices for self-monitoring and feedback, impacts on autonomy (ownership).	Final exercise is an interactive quiz exploring participants' experience of use of NoHoW Health Graphs. Tailored feedback is provided.

2.b. "My weight goal"	1) Reflect on ideal and acceptable weights (and where those originated/ mean to the person).<br><br>2) Understand the importance of setting self-relevant and optimal goals: Set weight-related goal	Self-regulation processes (monitoring, seeking feedback, awareness of standards and actual behavior, goal setting and planning)<br>- Competence<br>- Autonomy (ownership)<br>- Intrinsic (vs. Extrinsic) goals	- Discrepancy between current behavior and goal (BCT v1_1.6)<br>- Assist in setting optimal challenge (MBCT20)<br>-Help develop a clear and concrete plan of action (MBCT22)<br>-Clarify expectations (MBCT19)	- By reflecting on one's own standards regarding ideal weight outcomes and associated behaviors and the actual current situation, there is a perception on discrepancy that it is expected to lead to (1) reflect on the origins of those ideals weight outcomes (external vs intrinsic – linked or not to deep values/aspirations), (2) that the ideals/current levels may be related with the nature of the motives (autonomous vs controlled), and (3) prompt individuals to reflect and set and acceptable weight goals.	An initial text about "ideals" is presented, and users are asked to reflect on the source of their ideal weight target and define it.
			-Explore life aspirations and values (MBCT4)<br>-Provide a meaningful rational (MBCT5).<br>-Clarify expectations (MBCT19)	The fact that it is a personally salient goal (intrinsic, linked to deeper values) promotes perception of autonomy and guide individuals to reflect about acceptable and meaningful weight goals.	Next, participants do a quiz on autonomous and controlled sources for the weight they set when entered the NoHoW (I want to achieve this goal because…e.g. other want me to). Tailored answers are given.
			-Help develop a clear and concrete plan of action (MBCT22)<br>-Provide choice (MBCT6);<br>-Assist in setting optimal challenge (MBCT20).	By prompting individuals to reflect on specific behavior-related (exercise and diet) goals that are challenging but achievable – optimal goals (based on previous experience), can foster their confidence and perception of mastery in formulating and engaging in the necessary courses of action to achieve those goals (self-efficacy).	Later, users are asked if they want to lose more weight or maintain what they have lost and then set up their WLM goal with the support of NoHoW – participant weight graph is displayed.
			-Goal setting outcome (BCTv1_1.3); Goal setting behavior (BCTv1_1.1) and action planning (BCTv1_1.4).<br>-Assist in setting optimal challenge (MBCT20).<br>-Help develop a clear and concrete plan of action (MBCT22)<br>-Promote self-monitoring (MBCT23	Goal setting creates the mindset for engaging in the necessary self-regulatory processes to achieve goals (skills, strategies) and a standard against which, changes will be compared. Planning reinforces this process and links goal-directed responses to situational cues, therefore creating the conditions for goal pursuit	It is introduced the idea of a green zone of weight variation (planned weight variation) and yellow zone of weight variation (weight trajectory that needs attention).

Module 3 - Myths and facts
1 session

Sub-modules	Goals	Theoretical constructs	Intervention techniques	Rationale	General Description of Implementations
2.a. Myths and facts	1) Promote factual knowledge about energy balance-related behaviors (exercise and diet) and WLM.<br><br>2) Promote awareness of benefits and risks of multiple health behavior change (e.g. spill-over effects) – relation with sustainability.	-Competence<br>-Autonomous motivation	-Identify misconceptions about condition or behaviors<br>-Provide a meaningful rationale (MBCT5)<br>-Credible Source (BCTv1_ 9.1)	-Learning how to identify scientific credibility of weight management strategies, will contribute to increase confidence in changing behaviors, and minimizes risk of relapse/failure	Firstly, a video is presented about weight regain and the reasons behind it. It is highlighted the importance of having evidence-based knowledge about weight management.
			- Credible Source (BCTv1_ 9.1);<br>- Creating interest and value for health behaviors;<br>-Explore potential sources of pressure for behavior change (MBCT2)	. - Acknowledging which practices (behaviors) are more associated with WLM we promote users' increased knowledge on what is important for WLM in terms of behavior change (competence).	Users are presented with a quiz followed by a fact sheet with myths and facts on WLM and related behaviors (exercise and diet).

Module 4 – My healthy goals

4 sessions

Sub-modules	Goals	Theoretical constructs	Intervention techniques	Rationale	General Description of Implementations
4.a Doing it my way	1) Promote awareness of multiple choices around behavior changes and WLM at the long-term (there is no "right" way).<br>2) Finding individual interests and seeking enjoyment and personal meaning around health behaviors.	-Autonomy (perceived choice);<br>- Autonomous motivation;<br>- Competence; self-efficacy	-Discrepancy between current behavior and goal (BCT v1_1.6)<br>- Explore life aspirations and values (MBCT4)<br>- Facilitate autonomous goals or outcomes (MBCT7)<br>- Help develop a clear and concrete plan of action (MBCT22)<br>-Provide choices (MBCT6);<br>- Goal setting outcome (BCTv1_1.3)<br>- Offer constructive, clear, and relevant feedback (MBCT21);<br>-Provide choice (MBCT6)<br>-Clarify expectations (MBCT19)	By reflecting on one's own standards regarding ideal weight outcomes and associated behaviors and the actual current situation, there is a perception on discrepancy that it is expected to lead to (1) reflect on the origins of those ideals weight outcomes (external vs intrinsic – linked or not to deep values/aspirations), (2) that the ideals/current levels may be related with the nature of the motives (autonomous vs controlled).	A text about "choices" is presented. Users are asked to reflect on the source of their goals, and how to create a feeling of choice and a more sustainable source of motivation for weight management - autonomous versus controlled sources for the weight management efforts.<br><br>Participants practice with a quiz about different sources of motivation: "Please identify whether each reason is internal or external"). Tailored answers are given.
				By setting self-endorsed weight goals and ponder about the sustainability of the chosen activities participants will foster their autonomous motivation.	Next, an animation video explores the perception of choice and explains the differences about external and intrinsic ways to achieve individual goals and how we feel about them (e.g., "shoulds" vs "wants").<br>A final text depicts the importance of personally meaningful goals and congruence with deep values/aspirations.
				Prompts participants to reflect about the chosen activities to achieve their health goals, promoting self-regulatory capacity and/or self-efficacy, and give opportunity to acknowledge feelings and challenges of current weight management process.	Later, reflect on how well they are dealing with the activities they chose to attain their goals.

4.b. What do I need	3) Explore personal resources for engaging in health behaviors (e.g. skills).	- Competence; Self-efficacy<br>-Self-regulation capacity<br>- Autonomy (perceived choice)	- Discrepancy between current behavior and goal (BCT v1_1.6)<br>- Assist in setting optimal challenge (MBCT20).<br>-Provide choice (MBCT6);<br>- Goal setting outcome (BCTv1_1.3); Goal setting behavior (BCTv1_1.1)<br>-Help develop a clear and concrete plan of action (MBCT22)<br>-Clarify expectations (MBCT19)	By prompting individuals to reflect on specific behavior-related (exercise and diet) goals that are challenging but achievable – optimal goals (based on previous experience), can foster their confidence and perception of mastery in formulating and engaging in the necessary courses of action to achieve those goals (self-efficacy).	A Likert type scale question first engages participants to measure their level of confidence that they can maintain the activity(ies) that will help them to achieve their goals. Tailored answers are given to prompt reflection about confidence.<br><br>Next, a whiteboard video is presented about how to build up confidence (natural variations, sources of confidence, and relationship with motivation).
				The fact that it is a personally salient goal (intrinsic, linked to deeper values) promotes perception of autonomy. Goal setting creates the mindset for engaging in the necessary self-regulatory processes to achieve goals (skills, strategies) and a standard against which, changes will be compared.<br>Awareness of multiple choices for monitoring and feedback, impacts on autonomy (ownership).	A text summarises the importance of developing a menu of choices and realistic goals, proposing the use physical activity and weight graphs as well to keep track of progress.

**4.c. Physical activity goal**	1) Learn about self-monitoring and self-referenced goals, how to set specific goals<br><br>2) setting action plans, and reflect on individual options and preferences	- Self-regulation: monitoring, seeking feedback, awareness of standards and actual behavior, goal setting, action planning<br>- Competence, self-efficacy<br>- Autonomy (perceived choice an ownership)	- Self-monitoring of behavior (BCTv1_2.3) and of outcome (BCTv1_2.4);<br>- promote self-monitoring (MBCT23);<br>-Discrepancy between current behavior and goal (BCT v1_1.6);<br>-Provide choice (MBCT6)<br>- Action planning (BCTv1_1.4)<br>- Help develop a clear and concrete plan of action (MBCT22)<br>-Assist in setting optimal challenge (MBCT20)<br>-Clarify expectations (MBCT19)	Providing guidance in goal setting will ensure the suitability of goals and for that manner will positively impact feelings of competence and self-regulatory skills.	Participants are invited to set specific, meaningful, and realistic physical activity goals. A text provides guidance throughout the activity.
				By prompting individuals to develop their own plan of action promotes self-efficacy, commitment to their efforts and sense of ownership.<br>Planning reinforces this process and links goal-directed responses to situational cues, therefore creating the conditions for goal pursuit.	After setting personal goal, participants are introduced to Action Planning, i.e., an interactive and structured table with pending lists allows participants to specify “how”, “when”, for “how long”, “where”, and “with whom” they will perform the activity that will help them to achieve their behavioral goal.

**4.d Healthy eating goal**	1) Learn about self-monitoring and self-referenced goals, how to set specific goals<br><br>2) setting action plans, and reflect on individual options and preferences	- Self-regulation: monitoring, seeking feedback, awareness of standards and actual behavior, goal setting, action planning<br>- Competence, self-efficacy<br>- Autonomy (perceived choice an ownership)	- Self-monitoring of behavior (BCTv1_2.3) and of outcome (BCTv1_2.4);<br>- promote self-monitoring (MBCT23);<br>-Discrepancy between current behavior and goal (BCT v1_1.6);<br>-Provide choice (MBCT6)<br>- Action planning (BCTv1_1.4)<br>- Help develop a clear and concrete plan of action (MBCT22)<br>- Assist in setting optimal challenge (MBCT20<br>- Clarify expectations (MBCT19)	Providing guidance in goal setting will ensure the suitability of goals and for that manner will positively impact feelings of competence and self-regulatory skills.	Participants are invited to set specific, meaningful, and realistic physical activity goals. A text provides guidance throughout the activity.
				By prompting individuals to develop their own plan of action promotes self-efficacy, commitment to their efforts and sense of ownership.<br>Planning reinforces this process and links goal-directed responses to situational cues, therefore creating the conditions for goal pursuit.	After setting personal goal, participants are introduced to Action Planning, i.e., an interactive and structured table with pending lists allows participants to specify “how”, “when”, for “how long”, “where”, and “with whom” they will perform the activity that will help them to achieve their behavioral goal.

Module 5 - My goals and Values
2 sessions

Sub-modules	Goals	Theoretical constructs	Intervention techniques	Rationale	General Description of Implementations
**5.a. Shoulds and wants**	1) Prompt reflection on personal reasons for WLM (weight goals) and related-behaviors, by differentiating internal (autonomous) and external (controlled) motives, and its relation with sustained behavior change (sense of ownership).	Autonomous motivation vs - Controlled motivation<br>-Autonomy (ownership)<br>- Motivation sustainability	- Explore perspectives on condition or behavior (MBCT1);<br>-Acknowledge and respect perspectives (MBCT10)<br>-Provide a meaningful rationale (MBCT5)<br>-Facilitate autonomous goals or outcomes (MBCT7)<br>- Reflect-reframe on reasons and lasting changes (linked to sustainable practices)	- People have many different reasons (autonomous vs. controlled motives) for engaging or not engaging in health behaviors. These reasons matter for the sustainability of behavior change (exercise, diet) and WLM.	First, a text explains the different layers from controlled to more autonomous motivation and users are asked to fill in a list of "shoulds" and "wants".<br>A text clarifies how to distinguish "Shoulds" from "Wants" and why it matters to behavior change sustainability and weight management. Strategies are provided to promote autonomous behavior and intrinsic motivation.
**5.b Values and Motivation**	1) Explore sources of body image/ideal (e.g. societal norms, media, significant others), and its consequences (motivation, wellbeing).<br>2) Prompt reflection on how WLM and related-behaviors goals (and what has already been achieved) can be linked to deeper values and beliefs.	- Perceived social influences/ pressures related to weight<br>-Intrinsic (vs. extrinsic) goals<br>- life aspirations<br>-Autonomous motivation vs - Controlled motivation<br>- Motivation sustainability	- Explore life aspirations and values (MBCT_4)<br>- Values exploration (MI Taxonomy); Closed to valued self-identity (BCTv1_13.4);<br>-Clarify expectations (MBCT19)<br>-Explore ways of dealing with pressures (MBCT25)<br>-Explore intrinsic rewards (MBCT8)<br>-Provide a meaningful rationale (MBCT5)	By ranking life goals/values by importance participants will raise their awareness of what really matters for them in that specific moment of their lifes.<br><br>By linking those life goals and values with weight management goals, participants are able to redirect their efforts to more autonomous and intrinsic goals, and by doing that sustainability of those goal is reinforced.	Users are presented with a list of life goals/values and asked to select the most important to them. Then, they are asked to reflect on the link between these values and health behaviors and weight management. Then, they are asked to consider the nature of these values: intrinsic vs. extrinsic, and potential implications for long-term WLM and health behaviors.
			-Provide a meaningful rationale (MBCT5)<br>-Explore intrinsic rewards (MBCT8)	Providing clear examples of behavior change sustainability by comparing with renewable energy participant will learn the importance of choosing more autonomous and easily sustaining weight maintenance goals.	Next, the TK displays an image with a fictional science newsflash explaining these different types of motivations comparing them with sustainable energy (Fossil vs Renewal Energy).
			-Facilitate autonomous goals or outcomes (MBCT7)<br>-Provide a meaningful rationale (MBCT5)<br>-Explore life aspirations and values (MBCT4)<br>- Consideration of inter-goal influences (hierarchies)<br>- Valued self-identity (BCT 13.4)<br>- Values exploration (MI Taxonomy); Closed to valued self-identity (BCTv1_13.4);	Selecting self-relevant goals, consistent with the person's interests and values, can lead to better and long term behavior change, and foster need satisfaction (specially autonomy).	Participants are asked to choose up to 3 values that provide renewable energy (autonomous motivation). Participants are prompted to write down how their WM goals are congruent with their life values.

				By acknowledging discrepancy between present values and actual behaviors participants will be prompt to reflect what could be change to align weight managing goals within their life aspirations.	A picture illustrates the gap between personal values and weight management goals. A text highlights and reinforces the importance of having WM goals aligned with personal values and other meaningful goals (autonomous motivation).

Module 6 - Free and flexible
2 sessions

Sub-modules	Goals	Theoretical constructs	Intervention techniques	Rationale	General Description of Implementations
6.a Body Image	1.Identify functional and dysfunctional investment in body appearance (by exploring sources of body image/ideal (e.g. societal norms, media, significant others), and its consequences (motivation, well-being) and promote satisfaction with one's body, at any size.	-Intrinsic (vs. extrinsic) goals - life aspirations<br>- Autonomous vs. controlled motivation<br>- Autonomy (ownership)<br>- Rigid vs. Flexible behav regulation<br>- Modelling	-Facilitate autonomous goals or outcomes (MBCT7)<br>-Explore potential sources of pressure for behavior change (MBCT2)<br>- Reduce negative emotions (BCT 11.2)<br>- Explore ways of dealing with pressure (MBCT25)<br>- Provide a meaningful rationale (MBCT5)<br>- Explore intrinsic rewards (MBCT8)	Explore one's own standards regarding ideal body image and the nature of the feelings that arise from that, by prompting (1) reflection on the origins of ideal weight outcomes and body image (external vs intrinsic – linked or not to deep values/aspirations), (2) that the ideals/current body image may be related with the nature of the motives (autonomous vs controlled), (3) prompt individuals to reflect and set realistic weight goals and think about the effort they put in to accomplish these goals.	An introduction text helps participants to understand that ideals about body weight may be externally sourced and that this may impact the way they view their body.<br><br>Next, users are asked to fill a questionnaire about how they feel about their body, i.e, having self-centered and healthier perspectives about body image versus more externally oriented body views focused on negative feelings and appreciations of others. Tailored feedback is given immediately after.
			-Explore potential sources of pressure for behavior change (MBCT2)<br>- Explore ways of dealing with pressure (MBCT25)<br>- Reduce negative emotions (BCT11.2)<br>- Provide a meaningful rationale (MBCT5)	Two testimonies will provide real life based background about external body image vs self-centered perspective	Two testimonies with opposing backgrounds are presented to illustrate 1) external perspective of body image reporting negative feelings about weight management; 2) self-centered/autonomous perspective of body image in weight management, reporting positive feelings and enjoyment in challenging overall body function.
6.b Flexibility for sustainability	1) Explore functional and dysfunctional investment in body appearance, and promote satisfaction with one's body, at any size<br>2) Explore links between internal	-Intrinsic (vs. extrinsic) goals - life aspirations<br>- Autonomous vs. controlled motivation<br>- Rigid vs. Flexible behavior regulation<br>- Motivation sustainability	- Reduce negative emotions (BCT11.2)<br>- Provide a meaningful rationale (MBCT5)<br>- Facilitate autonomous goals or outcomes (MBCT7)<br>- Explore ways of dealing with pressure (MBCT25)	By identifying rigid thoughts users may try different approaches to weight maintenance and body image acceptance. Understanding the characteristics of a flexible mindset and providing specific tools to overcome negative self-talk, will have a positive impact in long-term weight management by providing emotional and self-regulatory support to probable set-backs (i.e., weight regain, over eating episodes)	A whiteboard animation depicts the importance of flexible thought and adjustable behavior regulations to attain weight goals. It also portrayed the idea of flexibility to cope with set-backs and to diminish negative emotions.

	(feels free and choiceful) and external (feels pressured) motives and eating and exercise regulation (e.g. rigid vs. flexible approach).				
			-Facilitate autonomous goals or outcomes (MBCT7)<br>- Explore ways of dealing with pressure (MBCT25)<br>- Reduce negative emotions (BCT11.2)<br>- Explore intrinsic rewards (MBCT8)<br>- Provide a meaningful rationale (MBCT5)	Opportunity to integrate new information by practicing with simple interactive activities relating to rigid and flexible thought.	A dichotomous questionnaire is presented to help participants to better discriminate rigid from flexible thinking (identify each of 8 sentences as rigid or flexible thinking).<br><br>Interactive exercise to provide useful technique to cope with a rigid mindset: (1) identify the rigid thought, (2) prompt reflection whether it is really true, (3) anticipate what to do/think when the rigid thought arise again.

Module 7- What gets in the way
2 sessions

Session	Goals	Theoretical constructs	Intervention techniques	Rationale	General Description of Implementations
**7.a.** Physical activity barriers	1) Identify challenges/barriers to current behavioral patterns and identify resources to increase capability to deal with them (for physical activity).<br><br>2) Identify strategies to deal with these barriers (coping plans) and to focus attention on how behavior changes serve other important life goals (for physical activity).	-Competence; self-efficacy<br>-Autonomous motivation<br>-Self-regulation processes: coping and persistence, relapse prevention.	-Address obstacles for change (MBCT18)<br>-Promote self-monitoring (MBCT23)<br>-Clarify expectations (MBCT19)<br>- Explore ways of dealing with pressure (MBCT25)<br>- Problem Solving (includes 'Relapse Prevention' and 'Coping Planning'; BCT 1.2)<br>- Reduce negative emotions (BCT 11.2)<br>-Assist	Positive experiences and successful coping plans foster perception of competence. Negative emotions related to potential failure are reduced.<br><br>Prompt participants to identify personal barriers to physical activity. Anticipate obstacles to plan ahead way to cope with them and prevent relapses.	Participant first read a testimony reporting the challenges he had to face during the weight loss process. It is introduced the idea of planning ahead of time difficulties that may potentially occur in the future what can be done to overcome those challenges.<br><br>In the next activity, participants are asked to choose 3 items from a pre-selected list of commonly reported physical activity barriers, and rate them in order from the most to the least challenging one.
				Defining coping plans provide structure and enhances self-efficacy. Participants will be more confident in their capacity to maintain physical activity goals and deal with pressures.	A text introduces examples of "coping planning" strategies to cope with participants barriers for physical activity.<br><br>An interactive web based app is presented to guide participants through "coping planning" with an "If-Then" sentence structure. A pending list provides the most common physical activity barriers ("other" is also a available) to help to define the "IF" part of the coping plan sentence. Next participants have a text box to write how they plan to cope with that chosen difficulty and with this they set the "Then" part of the sentence. The "If-Then" Coping Plan sentence will be saved and always available to update in the "Steps Tile" menu.

**7.b.** Healthy eating barriers	1) Identify challenges/barriers to current behavioral patterns, and identify resources to increase capability to deal with them (for eating behavior).<br><br>2) Identify strategies to deal with these barriers (coping plans) and to focus attention on how behavior changes serve other important life goals (for eating behavior).	-Competence; self-efficacy<br>-Autonomous motivation<br>-Self-regulation processes: coping and persistence, relapse prevention.	-Address obstacles for change (MBCT18)<br>-Promote self-monitoring (MBCT23)<br>- Explore ways of dealing with pressure (MBCT25)<br>- Problem Solving (includes 'Relapse Prevention' and 'Coping Planning'; BCT 1.2)<br>- Reduce negative emotions (BCT 11.2)	Positive experiences and successful coping plans foster perception of competence. Negative emotions related to potential failure are reduced.<br><br>Prompt participants to identify personal barriers to physical activity. Anticipate obstacles to plan ahead way to cope with them and prevent relapses.	Participant first read a testimony about the challenges the character had to face during the weight loss process. It is introduced the idea of planning ahead of time difficulties that may potentially occur in the future what can be done to overcome those challenges.<br><br>In the next activity it is asked that participants choose 3 items from a pre-selected list of commonly reported healthy eating barriers, and rate them in order from the most to the least challenging one.
				Defining coping plans enhances self-efficacy. Participants will be more confident in their capacity to maintain physical activity goals.	A text introduces examples of "coping planning" strategies to cope with participants barriers for healthy eating.<br><br>An interactive form of "coping planning" with an "If-Then" sentence structure is presented. A pending list provides the most common healthy eating barriers ("other" is also a available) to help to define the "IF" part of the coping plan sentence. Next participants have a text box to write how they plan to cope with that chosen difficulty and with this they set the "Then" part of the sentence. The "If-Then" Coping Plan sentence will be saved and always available to update in the "Steps Tile" menu.

Module 8 - My support system
2 sessions

Session	Goals	Theoretical constructs	Intervention techniques	Rationale	General Description of Implementations
8a. Social Support	1) Identify sources of social support and reflect on what they mean (e.g., pressured, conditional support vs. unconditional).	- Relatedness<br>- Perceived social influences / pressures related to weight<br>- Self-regulation processes (social coping and persistence, relapse prevention)<br>- Motivation sustainability	- Explore sources of support from others (MBCT24);<br>-Social support (emotional; BCT 3.3.)<br>- Explore ways of dealing with pressure (MBCT25)<br>- Social support (CALO-RE 29)<br>- Reduce negative emotions (BCT 11.2)<br>- Acknowledge feelings (MBCT11)	By differentiating what types of social support is expected from whom, participants will better cope with difficult situations of external pressure and learn to be aware of their feelings in these situations.	A brief text introduces the importance of social support in weight management efforts.<br><br>An interactive web-based activity explores the idea that some people are closer to us and may be defined as part of our "inner circle" but other people are more distant, that is, they are in our "outer circle" (a text box is available to allow participant to type names of their relatives).
				Explore different levels of relatedness by distinguishing who is in better position to give support to participants' weight management efforts.	After distinguishing the Inner and Outer circle persons of the participant's life, another web-based exercise helps them to suppose challenging social situations to their weight management goals. For each situation it is asked to identify whether it is something they already have or would like to have from the Inner circle or from the Outer circle and how useful and supportive that would be.
				Learning how to nurture a supportive social context participants' may reduce feelings of social influences, external pressure (controlled motivation).<br><br>The protective environment also strengthens competence, autonomy and relatedness, consequently, reducing negative emotions related to potential relapses.	A text describes the way social relationship may support or may hinder weight maintenance efforts. A graph bar depicts the evidence of the importance of having social support.<br><br>Interactive exercise using a visual analogue scale attached to an emoji to state whether the given examples of typical social interactions are felt as useful and supportive to the participants´ weight management goals.<br><br>The final text prompts participants to reflect about the differences of conditional social support and unconditional social support.

<table>
<tr>
<td rowspan="3">8b. Getting what I need</td>
<td rowspan="3">1) Increase skills in social support seeking and dealing with social / peer pressures (e.g. assertiveness).
2) Explore reaching out to others as a role model or source of support and expertise in WLM.</td>
<td rowspan="3">- Relatedness
- autonomous motivation
- Perceived social influences / pressures related to weight
- Self-regulation processes (social coping and persistence, relapse prevention)
- Motivation sustainability</td>
<td rowspan="3">-Explore sources of support from others (MBCT24)
-Social support (emotional; BCT 3.3.)
-Explore ways of dealing with pressure (MBCT25)
- Social support (CALO-RE 29)
-Encourage the person to be supportive towards others with a similar condition (MBCT9).</td>
<td>Participants identify differences between conditional support (controlled motivation) and unconditional support (autonomous motivation).

Communications strategies help to manage relationships to promote social supportive environments.</td>
<td>This session starts with a web based interactive activity to discriminate several social situations as conditional support (controlled) or unconditional support (autonomous).

Next, a set of texts are presented to explain other types of social support: emotional and instrumental. It is also introduced key aspects of communication and how participants can foster their social supportive environment. Examples of daily life situations are provided to clarify how participants may communicate their feelings in order to nurture a more supportive relationship with others.</td>
</tr>
<tr>
<td>Two testimonies model the importance of social support and it is highlighted that both supporter and supported benefit from the interaction.</td>
<td>Two testimonies are available to read. First one is an example of someone that helped a work colleague to become more active. And in the end, she found herself enjoying the new activities they were performing together. Second testimony is from someone who helped an old friend to start eating healthily. He shared some strategies that worked for him and after some time both were benefiting from each other’s skills and experiences.</td>
</tr>
<tr>
<td>This way participants will anticipate and learn how better cope with negative emotions associated with conditional social support.</td>
<td>The final text prompts participants to remember situations that they helped someone else and if it improved their eating habits or if they become even more active. It is also described how being supportive to others could be a good way to boost autonomous motivation.</td>
</tr>
</table>
