# Supplementary material for: A Theory- and Evidence-Based Digital Intervention Tool for Weight Loss Maintenance (NoHoW Toolkit): Systematic Development and Refinement Study
Source: J Med Internet Res. 2021 Dec 3;23(12):e25305. doi: 10.2196/25305 (PMC8686406; doi:10.2196/25305)
Supplement: Multimedia Appendix 10 [file jmir_v23i12e25305_app10.pdf]

Multimedia Appendix 10. Example of a questionnaire

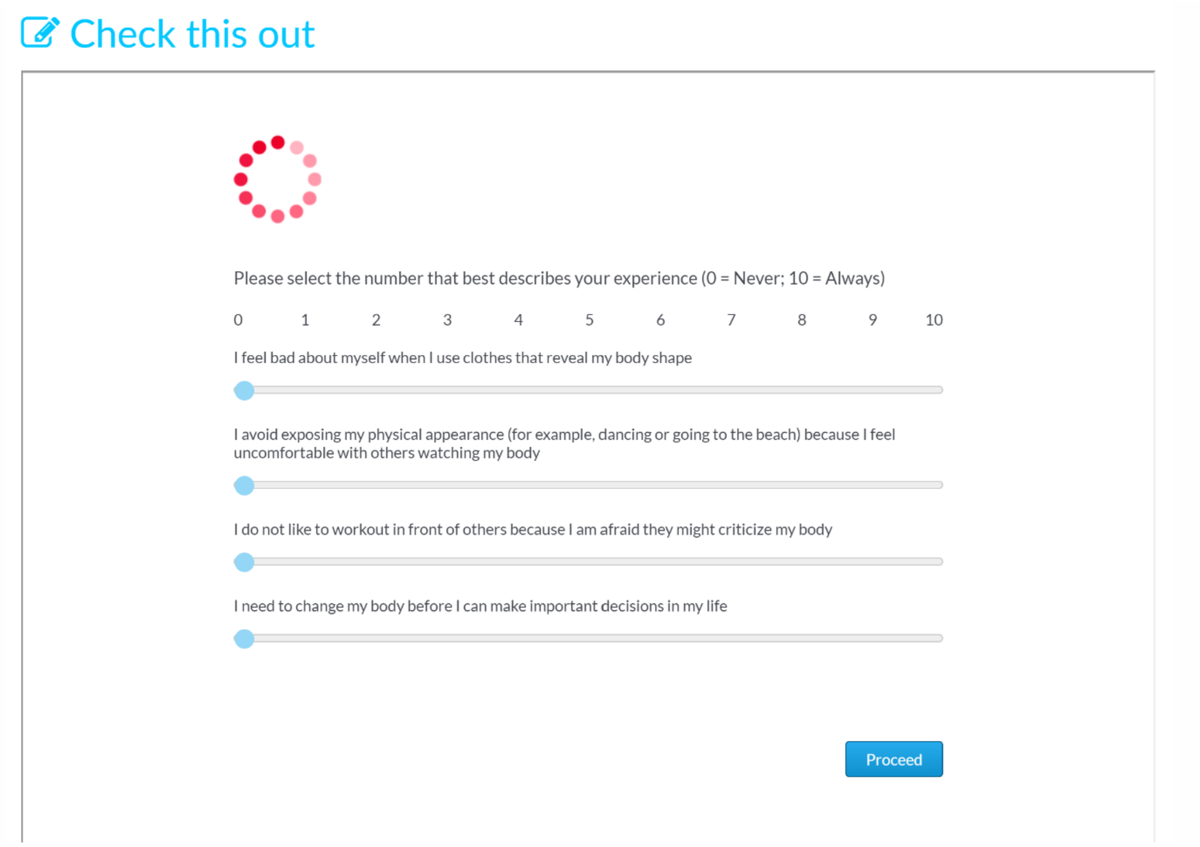

Check this out

Please select the number that best describes your experience (0 = Never; 10 = Always)

0 1 2 3 4 5 6 7 8 9 10

I feel bad about myself when I use clothes that reveal my body shape

I avoid exposing my physical appearance (for example, dancing or going to the beach) because I feel uncomfortable with others watching my body

I do not like to workout in front of others because I am afraid they might criticize my body

I need to change my body before I can make important decisions in my life

Proceed
