# Supplementary material for: A Theory- and Evidence-Based Digital Intervention Tool for Weight Loss Maintenance (NoHoW Toolkit): Systematic Development and Refinement Study
Source: J Med Internet Res. 2021 Dec 3;23(12):e25305. doi: 10.2196/25305 (PMC8686406; doi:10.2196/25305)

Multimedia Appendix 11. Example of a testimony

Testimony

Susan had a hard time fitting exercise into her busy family, but she found that exercising could also be a family activity. Here she talks about her experience of monitoring her weight and health-related behaviours as part of her weight loss journey.

Susan, 39 years old

"Looking back I have to say that one of the things that helped me the most to successfully lose weight was weighing myself regularly. At the beginning I would step on the scales daily. I still weigh myself regularly (at least once a week) to make sure I'm keeping it off.
In the early days, I also started the habit of monitoring and recording my diet every day. With time I realised it greatly helped me to achieve my diet goals and set new ones. It takes a bit of time and effort, but it really means that I stay on top of what I'm eating.
It's so easy to slip back into old patterns. My current goal is to increase my daily veggie intake!"

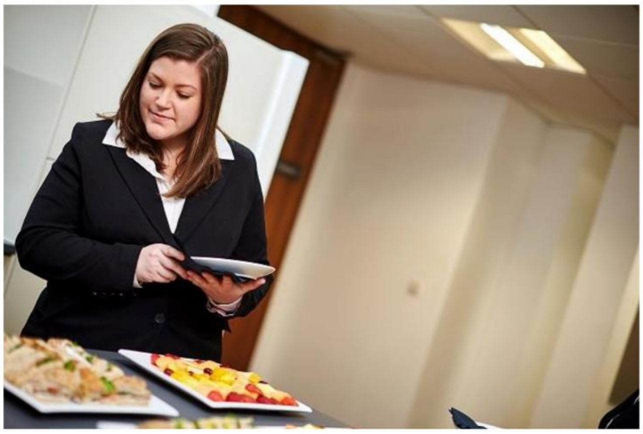

Supplement: Multimedia Appendix 11 [file jmir_v23i12e25305_app11.pdf]
